# Insufficient correctness of package inserts for psychotropic drugs in Germany

**DOI:** 10.1007/s00210-024-03430-y

**Published:** 2024-09-20

**Authors:** Adina Arning, Roland Seifert

**Affiliations:** Institute of Pharmacology, Hannover Medical School, Carl-Neuberg-Straße 1, 30625 Hannover, Germany

**Keywords:** Package insert, MGPCR antagonists, NE/5-HT enhancers, Comprehensibility, Potential for improvement

## Abstract

Package inserts often cause displeasure among patients as they are perceived as misleading and confusing. The aim of this study was to find out how comprehensible, complete, and truthful package inserts are formulated in Germany. 311 package inserts for antipsychotics (mGPCR antagonists) and antidepressants (NE/5-HT enhancers) from different manufacturers and dosages were analysed. The analysis criteria included the description of the effect, the warning of increased suicide risk, the explanation of interactions with co-medication, food and stimulants, as well as alcohol and the warning of impaired roadworthiness. In addition, the timeliness of the information regarding pregnancy and breastfeeding was checked and the symptoms mentioned under the topic of discontinuation symptoms and adverse drug reactions were compared. For most parameters, inconsistencies among various products, deficiencies and incorrect information were noted. This was particularly true for the items pregnancy and breastfeeding. Thus, package inserts for mGPCR antagonists and NE/5-HT enhancers need to be updated and standardised urgently. Such measures will reduce confusion among patients and increase drug adherence.

**Supplementary Information:**

The online version contains supplementary material available at 10.1007/s00210-024-03430-y.

## Introduction

In Germany, the addition of a package insert has been mandatory since 1978 (Schöne 2008). While the requirements of the German Medicines Act regarding the content have become increasingly detailed over the years, the general attitude of patients towards package inserts has hardly changed. More than 15 years ago, a study pointed out that package inserts were too long and illegible and therefore not understood. Even back then, this led to uncertainty on the part of patients (Nink and Schröder 2005). Although the German Medicines Act §11 has already been revised several times since the study, the suspicion remains that there has been no improvement in the package inserts in favour of patients.

Over the past 10 years, the percentage of people with outpatient diagnoses of mental disorders has increased by more than 10%. The greatest increases were seen among 11- to 17-year-olds and in 60- to 84-year-olds (Thom et al. 2024). Especially due to the Covid pandemic the awareness of mental health has gained importance. These two developments have resulted in an increase in the prescription of psychotropic drugs (Ludwig et al. 2024). Therefore, we decided to analyse the package inserts of psychotropic drugs.

In recent years, there have been repeated reports that certain preparations are temporarily unavailable, and the number of supply bottlenecks is increasing (Menner 2024). Thus, patients have to change their preparations and sometimes even their active ingredients. For patients, it is often not only the design of the packaging that changes, but also the package insert. The package insert, which is intended to provide patients with thorough and comprehensive information about their preparation, now leads to confusion again. And what happens if the package inserts of the individual manufacturers contain different or even incorrect information? In the worst case, incorrect information can put the life of the patient and even others at risk.

Our previous work analysed package inserts on formal criteria. We showed that the criteria of the Readability Guideline are not met and that intelligibility of the package inserts has to improve (Arning and Seifert 2023). In or present study we analysed the correctness of pharmacological information of package inserts of psychotropic drugs in Germany.

## Material and methods

**Fig. 1:**
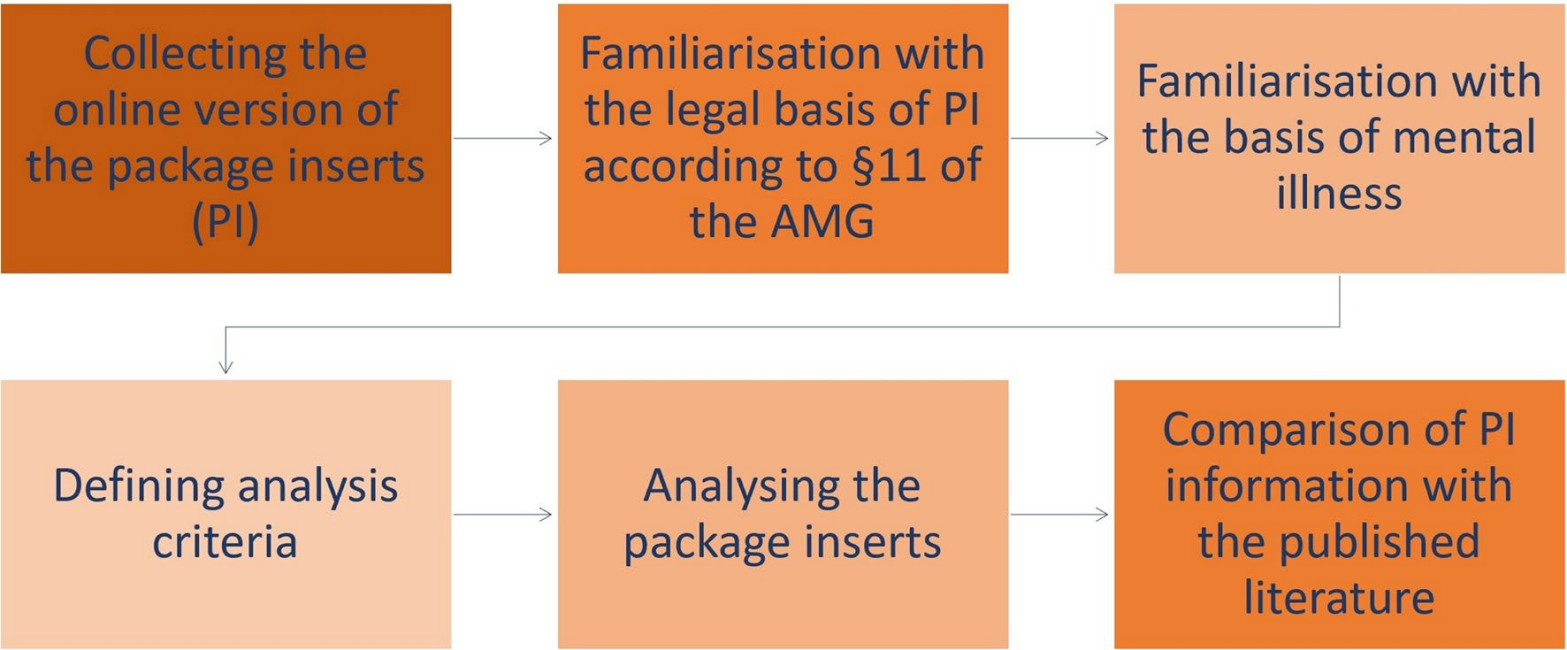
Flowchart picturing the work process for this article

A total of 311 package inserts (PI) for mGPCR antagonists, generally known as antipsychotics, and NE/5-HT enhancers, generally known as antidepressants, were analysed. The following paper uses the new mechanistic terminology required for this journal (Seifert and Schirmer 2020). Of the mGPCR antagonists, haloperidol, clozapine, melperone, olanzapine, opipramol, quetiapine, and risperidone were analysed, and of the NE/5-HT enhancers, moclobemide, venlafaxine, citalopram, escitalopram, and sertraline. The prerequisite for selection was that the active substance was also included in the product range of the reference manufacturer a. For each active ingredient, up to the top 5 other manufacturers, that were mentioned in the “Arzneiverordnungsreport (Drug Prescription Report)” (Ludwig et al. 2021) were used for comparison, from which all corresponding dosages and forms of administration were then taken into account. This cumulates to a total of 311 PIs, of which none were excluded. Of the 311 PIs, 225 PIs can be categorised as mGPCR antagonists and the remaining 86 PIs as NE/5-HT enhancers (Table 1, Fig. S1). Table 1 shows the individual active substances analysed in the two groups of psychotropic drugs, with their number of manufacturers and preparations. Each preparation was assigned an identification number; the range of identification numbers for an active substance is listed in the table. For data protection reasons, the individual manufacturers are not named, so they were coded with a letter code from a to u. The coded manufacturers of an active substance are listed in the last column of the table (Table 1) A more detailed list can be found in the supplement (Table S1). The flowchart roughly outlines the work process for this article (Fig. 1). The analysis was carried out by comparing the respective PIs with each other and by checking the information content for correctness and completeness using the current medical scientific literature. The analysis criteria used were taken from the requirements of the German Medicinal Products Act §11.

**Table 1:** Distribution of numbers of manufacturers and package inserts as well as identification numbers and coded manufacturers for each active ingredients

Psychotropic drugs	Active ingredient	Number of manufacturers	Number of preparations	Identification number	Manufacturers (coded)
**mGPCR antagonists**			**225**		
mGPCR antagonist	Haloperidol	3	23	1–23	a, b, k,
mGPCR antagonist	Clozapine	6	22	24–45	a, c, f, j, s
mGPCR antagonist	Melperone	6	21	46–66	a, b, c, d, e,
mGPCR antagonist	Olanzapine	6	45	67–111	a, b, c, h, i, j,
mGPCR antagonist	Opipramol	5	11	112–122	a, b, c, d, m
mGPCR antagonist	Quetiapine	6	65	123–187	a, b, c, d, f, g,
mGPCR antagonist	Risperidone	6	38	188–225	a, c, d, e, o, u
**NE/5-HT enhancers**			**86**		
NE/5-HT enhancer	Moclobemide	3	6	226–231	a, b, c
NE/5-HT enhancer	Venlafaxine	5	19	232–250	a, b, d, e, n
NE/5-HT enhancer	Citalopram	6	24	251–274	a, b, c, d, e, r
NE/5-HT enhancer	Escitalopram	6	25	275–299	a, f, i, j, l, t
NE/5-HT enhancer	Sertraline	6	12	300–311	a, c, g, h, p, q

Firstly, it was determined how the effect of the preparation is explained and whether the same indications are listed for each active ingredient regardless of the manufacturer. A comparison was then made of how the increased risk of suicide due to different efficacies is communicated in the corresponding PI.

In addition, the PIs were analysed about their information on interactions with co-medication, food and stimulants. Special aspects such as fertility, conception, pregnancy, and breastfeeding phases were compared with the information provided by the Pharmacovigilance and Advisory Centre for Embryonal Toxicology (Embryotox). Embryotox is a website dedicated to assessing the tolerability of drugs during pregnancy and breast-feeding (Embryotox Redaktion, a). About driving ability, it was analysed whether the information was correct and whether the effects were presented equally clearly in all PIs.

As the correct use of the medication is of eminent importance in everyday life, it was checked whether and how it is mentioned that various symptoms can occur in the event of sudden discontinuation. It was also determined whether the adverse drug reactions (ADRs) are labelled as ADRs as recommended or with the less precise term “side effects”. It was also checked whether the frequency of ADRs was specified and whether these were consistent in the respective PIs. Finally, a comparison was made as to which contact points were specified for reporting further ADRs.

## Results and discussion

### Effect

**Fig. 2:**
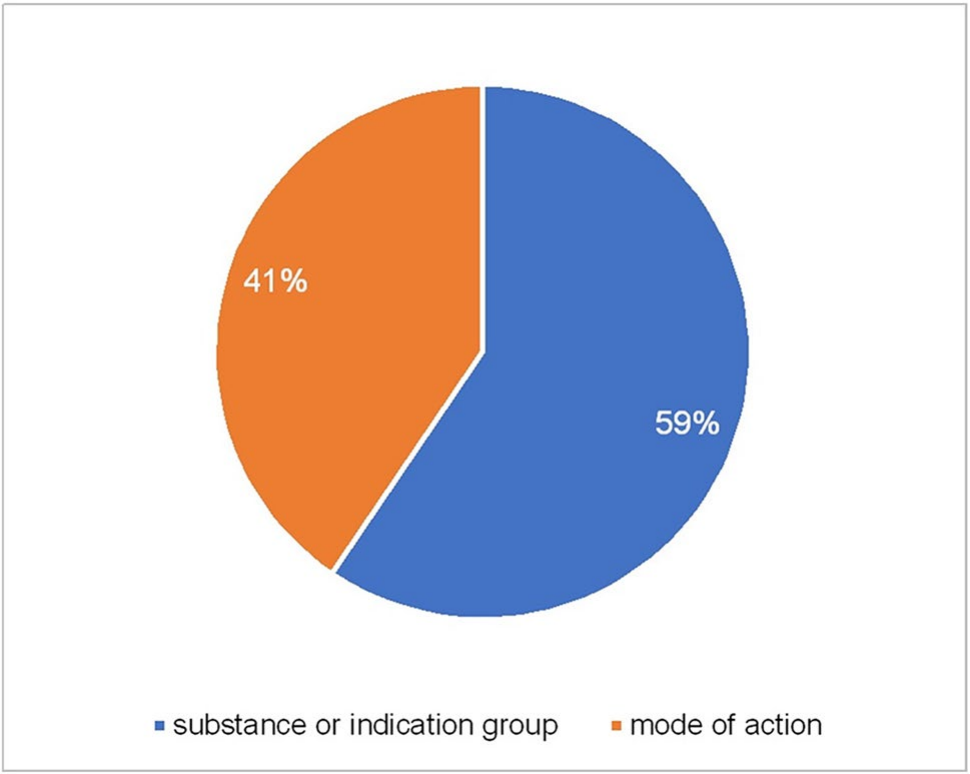
Effect-overall. This diagram shows the distribution of all 311 PI between PI in which the substance or indication group is mentioned (blue) and PI with the mode of action mentioned (orange)

**Fig. 3:**
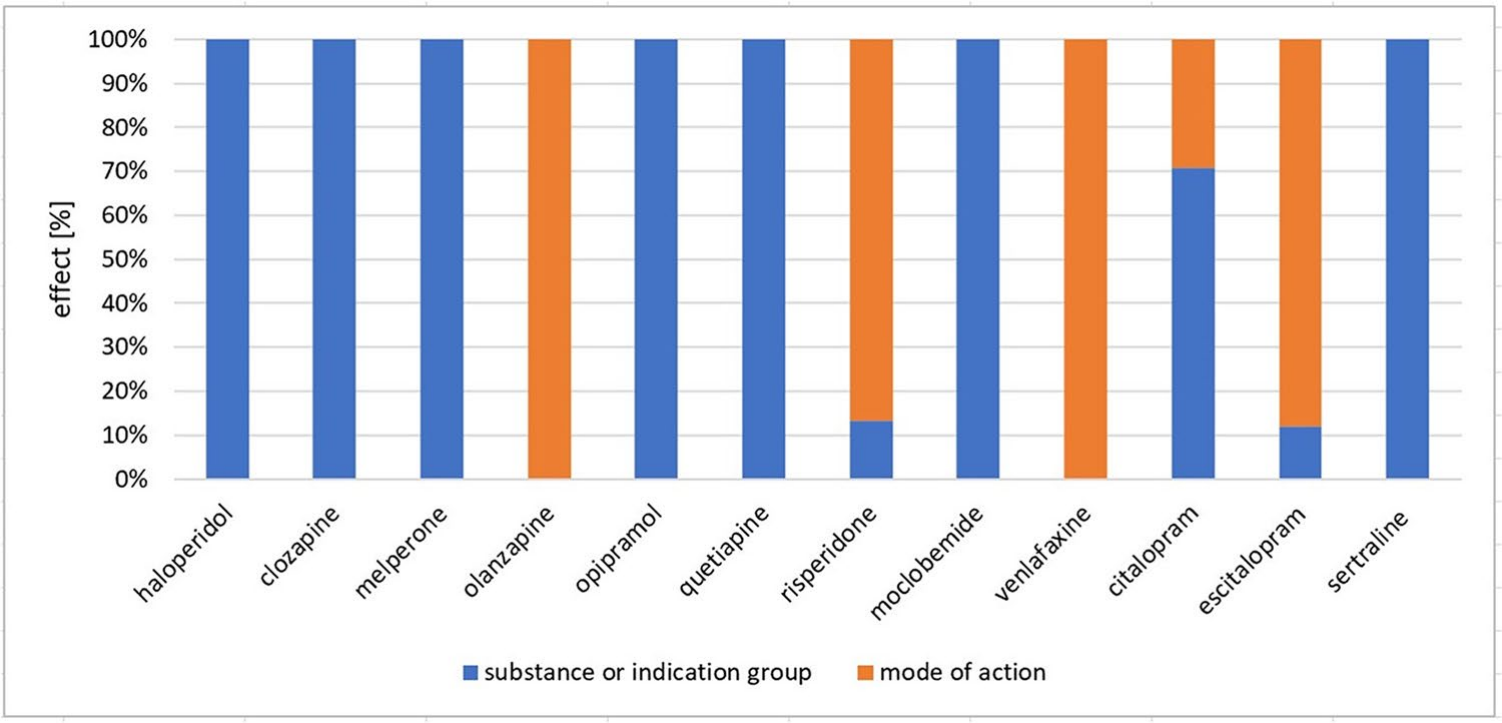
Effect of the respective drugs. This diagram shows how many PIs contain a description of the mode of action (orange) plotted against the active substances, as well as how many PIs only contain the substance or indication group (blue) plotted against the active substances

Paragraph 11 of the German Medicinal Products Act requires that either drug class or indication group or mechanism of action (Bundesministerium der Justiz 2019) be stated in the package insert. All 311 package inserts examined fulfil this requirement. In 59% (185 package inserts in absolute terms) only the substance or indication group is mentioned and in the remaining 41% (126 package inserts in absolute terms) the mode of action is also described (Fig. 2). However, this is not a detailed explanation, but just a very rough outline in 2 to 3 sentences. Whether the substance group or the effect, meaning the mode of action is stated, is usually standardised within a substance class. Exceptions to this are the active substances risperidone, citalopram, and escitalopram. For the PIs of the mGPCR antagonists haloperidol, clozapine, melperone, opipramol and quetiapine, the mode of action is mentioned, but not the substance group. In the case of olanzapine, both are mentioned (Fig. 3). The PIs of risperidone explain the mode of action of the substance in 87% (identification numbers 188–220), the remaining 13% (identification numbers 221–225) are limited to naming the substance group (Fig. S2). The modes of action of moclobemide and sertraline are not mentioned in the NE/5-HT enhancers, whereas they are mentioned for venlafaxine. 29% of the PIs of citalopram (identification numbers 251–254 and 259–262) contain an explanation of the mode of action, and 71% (identification numbers 255–258 and 263–274) contain only a mention of the substance or indication group (Fig. S3), 88% of the PIs of escitalopram (identification numbers 275–292 and 296–299) contain an explanation of the mode of action and 12% (identification numbers 293–295) contain only a mention of the substance or indication group (Fig. S4). It is striking that pharmacologically very similar drugs differ significantly in PIs (Fig. 3, Fig. S3, Fig. S4).

### Suicide risk

**Fig. 4:**
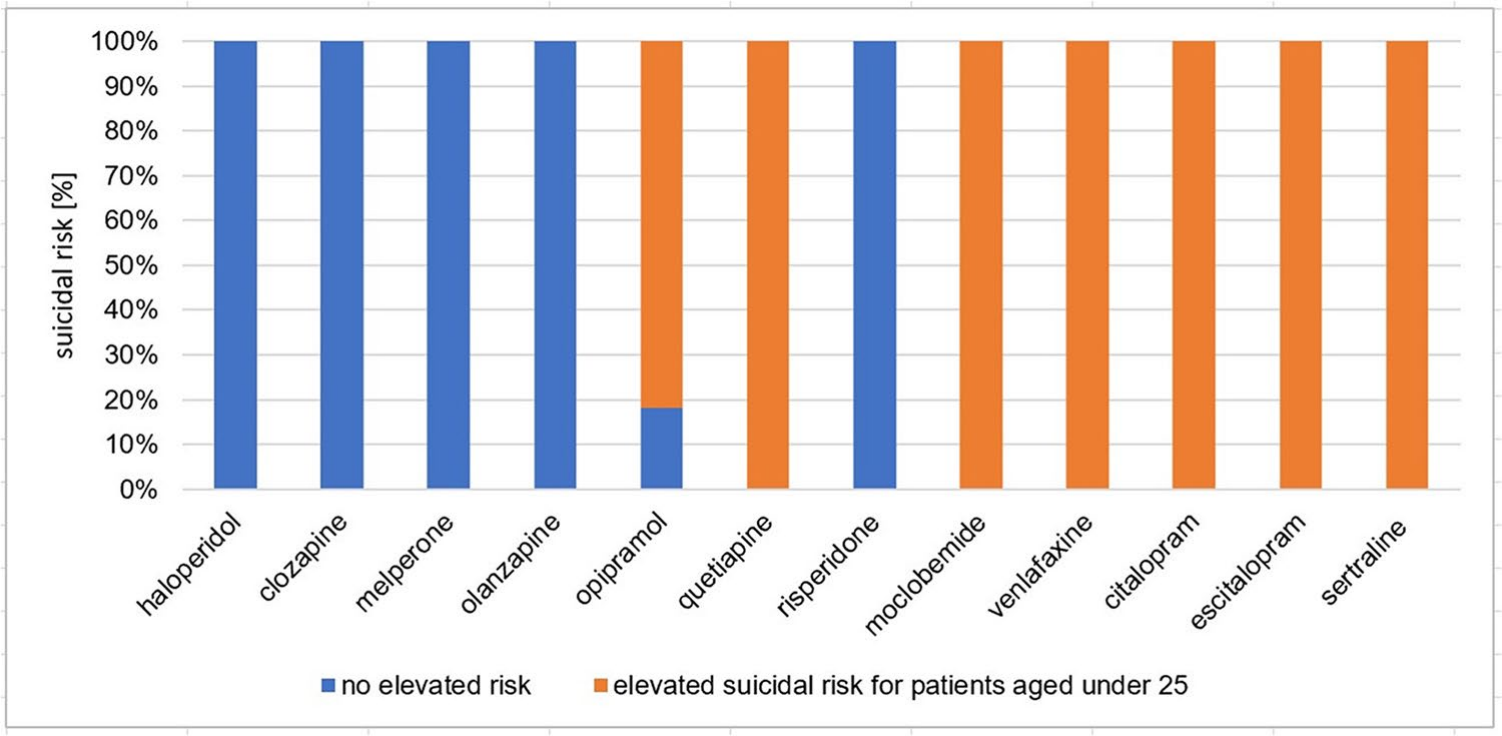
Suicidal risk. The diagram shows the suicidal risk in regard to the respective drugs. It distinguishes between active substances with no elevated risk (blue) and active substances for which an elevated suicidal risk is mentioned in the PI (orange)

“The suicide rates among people with serious mental illness [are already …] high” (Fu et al. 2023). Treatment with NE/5-HT enhancers can further increase this risk, as the various effects often only occur in a staggered manner when a new patient is prescribed the drug. As a rule, there is an increase in drive at the beginning and only after a time delay does the mood improve. The patient is therefore particularly at risk during this initial time interval and must be explicitly informed about this. No increased risk has been proven to date for treatment with mGPCR antagonists, but the extent to which a potential risk is pointed out was also examined. The analysis points out that 43% of all PIs analysed refer to an increased risk of suicide. In the other 57%, the topic is not mentioned. 100% of the PIs of NE/5-HT enhancers and 33% of the PIs of mGPCR antagonists refer to the increased risk (Fig. 4). Under 25-year-olds and patients who have already shown suicidal or auto-aggressive behaviour in the past are mentioned as particularly suicidal risk in all PIs of NE/5-HT enhancers and in those of quetiapine. If one searches the literature for the suicide risk associated with quetiapine use, “there is no evidence of treatment-emergent suicidality with quetiapine” (Weisler et al. 2014).

### Interactions with co-medication

Many PIs often include a long list of medications with which the preparation may interact. This fact often leads to uncertainty on the part of the patient. The patient may not be aware that many of the drugs listed are hardly relevant on the German market or in some cases are no longer authorised and, therefore, no longer pose a risk. The 55 irrelevant drugs mentioned belong, among others to the group of antibiotics (antibacterial drugs), anticoagulants, antihypertensives, diuretics, and psychotropic drugs (Table S2). The drugs are not listed in the drug prescription report of 2022 and 2023 or have no prescriptions (Ludwig et al. 2023).

In addition to the many drugs that are irrelevant, there is also at least one spelling mistake when mentioning the name of an active ingredient. For example, “thioridazine” becomes “thiorizadine”. A patient who does not recognise “their” medication could be at risk from potential interactions.

### Interactions with food and stimulants

In addition to interactions with co-medications, interactions with food and stimulants can also occur (Table 2). Whether and which interactions are to be expected is standardised in the PI of an active substance. According to the PI, there are no interactions with the drugs haloperidol, olanzapine, opipramol, venlafaxine, citalopram and escitalopram. As stated in the PIs clozapine, on the other hand, interacts with both caffeine and tobacco. The plasma concentrations are increased by caffeine consumption. This means that dose changes of clozapine may be necessary after a change in the usual caffeine intake (Kämmerer 2011). Tobacco consumption, on the other hand, leads to enzyme induction, which is associated with a faster breakdown of clozapine. The serum concentrations of clozapine achievable with a standard dose are considerably reduced in smokers and there may be a total loss of efficacy. An increase in dose is therefore necessary in smokers. Quitting tobacco smoking can lead to a sharp increase in the plasma level of clozapine. Patients should therefore be sure to inform their doctor of any plans to stop smoking. In such cases, the dose of clozapine should be reduced accordingly (Kämmerer 2011). Although the PI of clozapine indicates that a change in smoking behaviour can have an effect, the exact course of events is not explained, so that it remains unclear when and whether an under- or overdose can occur. For the intake of melperone, the PI does refer to interactions with milk, coffee, and tea, but this information has not been substantiated by scientific articles (search keywords on PubMed: melperone AND milk, melperone AND coffee, melperone AND tea). Quetiapine and sertraline are known to interact with grapefruit via cytochrome P450 3A4 metabolism. “Grapefruit juice has been shown to be an inhibitor of CYP3A4 [… and] there are many reports suggesting that grapefruit juice can inhibit the pharmacokinetics of drugs that are metabolized by CYP3A4. [A corresponding] increase [in] the plasma level” can thus be associated with an overdose (Ueda et al. 2009). When taking risperidone, the PIs differentiate between the solution and tablet form about the interaction. The tablets do not interact with food and stimulants, whereas the solution interacts with tea. The tea catechins, especially epifallocatechinallate (EGCg), form insoluble complexes with the risperidone solution, which reduce the efficacy of the drug (Goromaru et al. 2023; Ikeda et al. 2010, 2012). According to PI, the active ingredient moclobemide interacts with tyramine-rich foods such as mature cheese or red wine. Although the “hypertensive crisis after ingestion of tyramine-rich food [… only] becomes clinically relevant from a moclobemide dosage above 900 mg/d” (Bonnet 2003) or in combination with other drugs that interact with tyramine-rich food, the interaction should not be challenged regardless of the dosage.

**Table 2:** Information of interactions of psychotropic drugs with food and stimulants in PIs substantiated with information found on PubMed

Active substance	Information in PI	Consequences	Evidence/source
Haloperidol	No interaction	–	–
Clozapine	Caffeine, tobacco	1) Caffeine increases clozapine plasma concentration 2) Tobacco leads to enzyme induction ➔ faster metabolization of clozapine	Kämmerer (2011)
Melperone	Milk, coffee, tea	Unclear	No evidence
Olanzapine	No interaction	–	–
Opipramol	No interaction	–	–
Quetiapine	Grapefruit juice	Inhibition of CYP3A4 ➔ increased efficacy of the drug	Ueda et al. (2009)
Risperidone	Tea	Tea catechins form complexes ➔ reduced efficacy of the drug	Goromaru et al. (2023), Ikeda et al. (2010), Ikeda et al. (2012),
Moclobemide	Tyramine-rich food	Hypertensive crisis	Bonnet (2003)
Venlafaxine	No interaction	–	–
Citalopram	No interaction	–	–
Escitalopram	No interaction	–	–
Sertraline	Grapefruit juice	Inhibition of CYP3A4 ➔ increased efficacy of the drug	Ueda et al. (2009)

### Alcohol (ethanol)

**Fig. 5:**
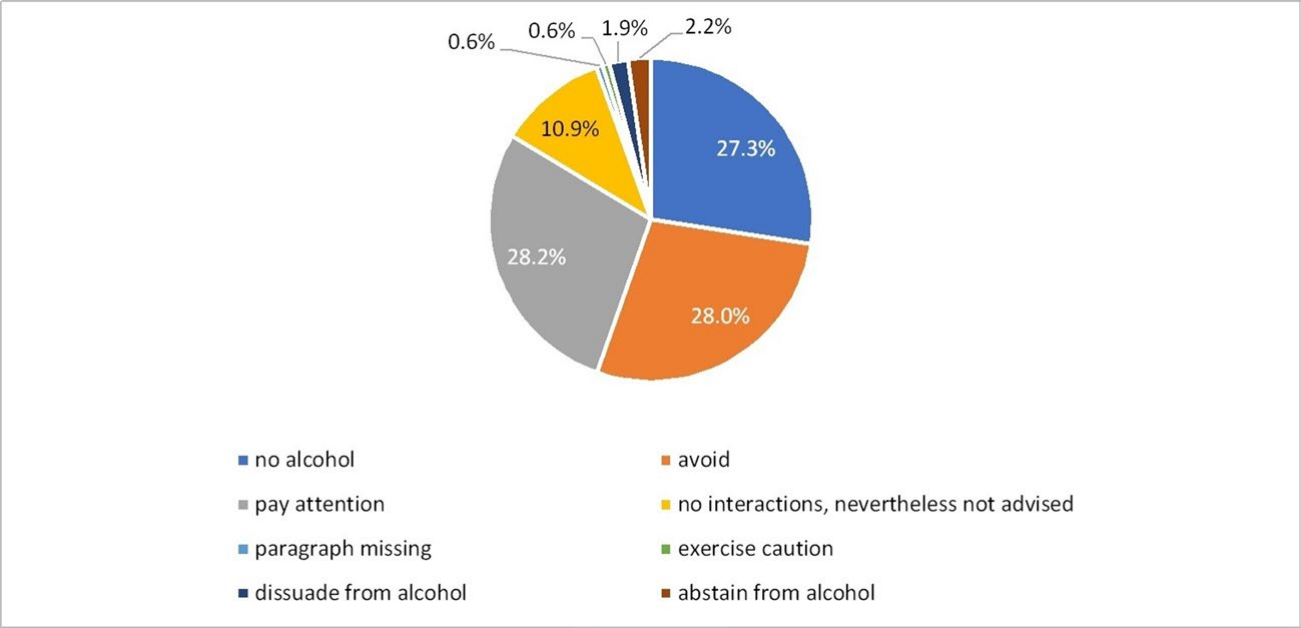
Various general formulations that warn against the additional intake of alcohol. This diagram shows the distribution of different formulations concerning the warning against the additional intake of alcohol during the therapy with psychotropic drugs

Interactions between the psychotropic drug and alcohol are generally always warned against, regardless of scientific evidence. The only difference is the urgency of the wording. The information in the package inserts for haloperidol, clozapine, olanzapine, melperone, quetiapine, risperidone, venlafaxine, and escitalopram is standardised regardless of manufacturer and dose. For opipramol, moclobemide and sertraline, the information provided by the individual manufacturers varies slightly but is independent of the dose. The same applies to venlafaxine, where there is an additional warning about extreme tiredness and unconsciousness. For the intake of citalopram, different information is given even for different dosages from one manufacturer. In 27.97% of PIs, it is advised to avoid alcohol, and in 28.20% of PIs, it is recommended to “pay attention” to it. According to 27.33% of PIs, the combination of alcohol with psychotropic drugs is prohibited. In 10.89% of the PIs, it is stated that no interactions are to be expected, but that the combination should nevertheless be avoided. Other formulations are “abstain” (2.24%), “advise against” (1.92%) or “give with caution” (0.64%). Only in 0.64% of the package inserts is the section on interaction with alcohol completely missing (Fig. 5, Fig. S5).

The interaction between alcohol and psychotropic drugs occurs either via pharmacodynamics or pharmacokinetics (Weathermon and Crabb 1999). However, no information on mechanisms of action can be found for the drugs melperone, opipramol, risperidone, and escitalopram. Haloperidol, olanzapine, citalopram, and sertraline interact via pharmacodynamics. For olanzapine, it has been proven that the combination with alcohol increases the depressant effect on the central nervous system, which can ultimately lead to a drop in oxygen saturation (Callaghan et al. 1999; Wilson et al. 2012). The combination of alcohol with citalopram or sertraline can result in changes in EEG waves (Pietrzak and Czarnecka 2003). Clozapine, quetiapine, moclobemide, and venlafaxine interact with alcohol via the pharmacokinetic mechanism. In the case of clozapine, “the concomitant use of alcohol is associated with a significant[ly potentiated] risk of severe adverse reactions” (Monroy-Jaramillo et al. 2022). In contrast, the combination of quetiapine with alcohol leads to a decrease in the serum level of the drug and to an increase in the effect of alcohol (Aburamadan et al. 2021; Gujjar et al. 2016). For moclobemide and venlafaxine, it is known that the interaction is pharmacokinetic, but no or only a few specific interactions with alcohol are mentioned (Tiller 1990; Troy et al. 1997; Uzbay et al. 1995).

### Pregnancy and sexual disorders

The reproductive topics covered in the PI relate to pregnancy, breastfeeding, fertility, and sexual dysfunction. Although the information in the PI is standardised, it is very general and therefore appears to be more of a legal safeguard. In addition, they do not correspond to the current scientific status of Embryotox. Embryotox was founded in 1988 and is dedicated to the professional exchange and harmonisation of counselling procedures as well as the scientific evaluation of exposed pregnancies since 1990 (Embryotox Redaktion, n). In 2004, the advisory centre became the Pharmacovigilance and Advisory Centre for Embryonal Toxicology (Embryotox Redaktion, n). The internet portal used was developed in 2008 and focuses on drug therapy during pregnancy and breastfeeding. The information on the respective active substance pages may differ from the information in the Information for healthcare professionals, the Red List and on the package leaflet. They take into account relevant study results from specialist journals as well as the current state of discussion in relevant professional associations (Embryotox Redaktion, a).

**Fig. 6:**
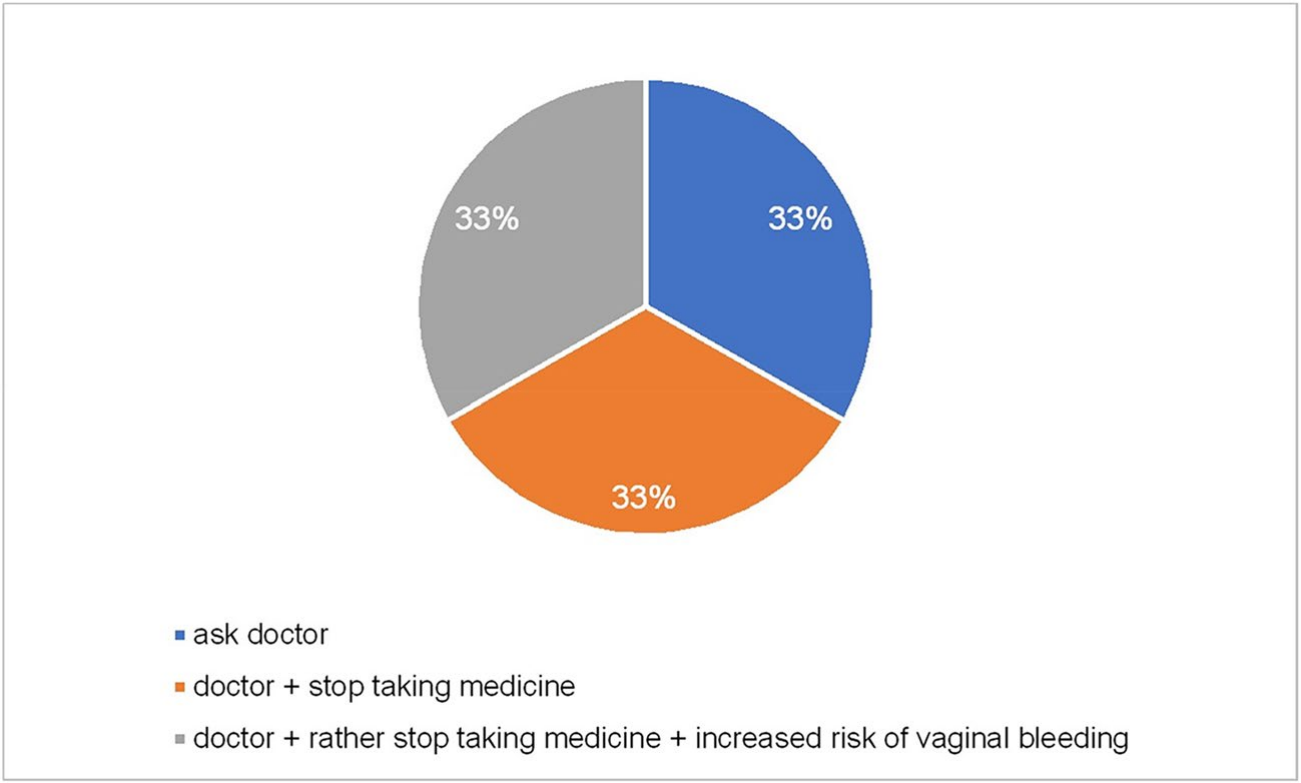
Package insert recommendations during pregnancy—overall. This diagram shows the distribution of different recommendations given in all 311 PIs for the time during the pregnancy

About pregnancies, the PIs for clozapine, olanzapine, risperidone and moclobemide only advise informing the doctor. This information, with the addition that the medication should be discontinued, can also be found in the PIs for haloperidol, melperone, opipramol, and quetiapine. The PIs for venlafaxine, citalopram, escitalopram, and sertraline advise to inform your doctor and discontinue the medication. There is also a warning about vaginal bleeding. In addition to the paragraph on pregnancy itself, all PIs, except those for opipramol and moclobemide, warn that continuous use of the medication can lead to withdrawal symptoms in the newborn within 24 h of delivery. These would then manifest themselves in the form of drowsiness, agitation, drinking difficulties and breathing difficulties, as well as hyper- and hyporeflexia (Fig. 6, Fig. S6).

Embryotox provides much more specific instructions than the package inserts. Haloperidol may be taken during pregnancy if no more tolerable antipsychotics, such as quetiapine or risperidone, can be considered (Embryotox Redaktion, e). In the case of clozapine, it is not necessary to switch patients who are well-controlled. However, readjustment should be carried out with better-proven mGPCR antagonists such as quetiapine, possibly risperidone or, if necessary, haloperidol (Embryotox Redaktion, c). If melperone was taken before pregnancy, a switch should be made to a better-proven sedative. Promethazine and, in the case of sleep disorders, amitriptyline, and diphenhydramine are suitable for this purpose (Embryotox Redaktion, f). The use of olanzapine can also be continued in well-adjusted patients, but a new adjustment to this medication should not be made during pregnancy, but rather started with quetiapine. In general, quetiapine, risperidone and haloperidol are better alternatives during pregnancy (Embryotox Redaktion, h). Opipramol therapy can be continued in well-adjusted patients, but an alternative medication should be chosen depending on the indication in the case of a new adjustment (Embryotox Redaktion, i). The mGPCR antagonist quetiapine can be taken in any case (Embryotox Redaktion, j). Treatment with risperidone may be continued in well-adjusted patients, but should be replaced by quetiapine in new patients (Embryotox Redaktion, k).

According to Embryotox, the use of NE/5-HT enhancers may generally be continued during pregnancy. When taking moclobemide, a switch should be urgently prevented. However, a readjustment, especially before a planned pregnancy, should be made directly with an active substance of the tricyclic antidepressants (TCA) or the selective serotonin reuptake inhibitors (SSRI) (Embryotox Redaktion, g). The prescription of venlafaxine does not have to be changed, but the new setting should rather be with citalopram or sertraline (Embryotox Redaktion, m). The SSRI escitalopram may continue to be taken during pregnancy, although citalopram and sertraline are also better alternatives here. According to Embryotox, the latter is the drug of choice for treating depression during pregnancy anyway and should therefore not be changed (Embryotox Redaktion, b; Embryotox Redaktion, d; Embryotox Redaktion, l).

Whenever psychotropic drugs are taken during pregnancy, close psychiatric and gynaecological monitoring should be carried out. When taking clozapine, olanzapine, quetiapine and risperidone, the risk of gestational diabetes must be considered. In addition, close ultrasound examinations, especially in the first trimester, should document unremarkable foetal development. If possible, the delivery should take place in a clinic with a neonatology unit, as postnatal adjustment disorders cannot be ruled out (Embryotox Redaktion, b; Embryotox Redaktion, c; Embryotox Redaktion, d; Embryotox Redaktion, e; Embryotox Redaktion, f; Embryotox Redaktion, g; Embryotox Redaktion, h; Embryotox Redaktion, i; Embryotox Redaktion, j; Embryotox Redaktion, k; Embryotox Redaktion, l; Embryotox Redaktion, m). When taking mGPCR antagonists, adaptation disorders can manifest themselves in the form of neurological, gastrointestinal, respiratory, or extrapyramidal-motor symptoms; very rarely, seizures can also occur. Due to the risk of agranulocytosis, the use of clozapine requires additional blood tests of the newborn child (Embryotox Redaktion, c; Embryotox Redaktion, e; Embryotox Redaktion, f; Embryotox Redaktion, h; Embryotox Redaktion, i;Embryotox Redaktion, j; Embryotox Redaktion, k). Taking the NE/5-HT enhancers moclobemide and venlafaxine can also lead to neurological, gastrointestinal, and respiratory disorders in the newborn after birth. In rare cases, the previous intake of venlafaxine can also lead to seizures or myoclonus in the newborn. In general, the use of SSRIs can lead to hyperexcitability, tremor, drinking disorders, respiratory distress syndrome, hypoglycaemia and sleep or behavioural abnormalities in newborns. It is therefore advisable to reduce the dose of the respective NE/5-HT enhancer one to two weeks before delivery and to increase it again postpartum to minimise withdrawal symptoms in the newborn as much as possible (Embryotox Redaktion, b; Embryotox Redaktion, d; Embryotox Redaktion, g; Embryotox Redaktion, l; Embryotox Redaktion, m). Table 3 shows a comparison of the information given in the package inserts and by the Embryotox website.

**Table 3:** Recommendations concerning treatment during pregnancy given in the package inserts versus the recommendations given by Embryotox including their alternative drug choices

Active ingredient	Information given in package insert	Information given by Embryotox	More suitable alternative given by Embryotox
Haloperidol	Ask doctor + stop taking medicine	Can be prescribed, if no better tolerated antipsychotic is possible (Embryotox Redaktion, e)	Quetiapine, risperidone (Embryotox Redaktion, e)
Clozapine	Ask doctor	Readjustment with better-proven antipsychotics, no change is necessary for well-adjusted patients (Embryotox Redaktion, c)	Quetiapine, risperidone, haloperidol (Embryotox Redaktion, c)
Melperone	Ask doctor + stop taking medicine	Readjustment with better-proven antipsychotics (Embryotox Redaktion, f)	Promethazine, for sleep disorders: diphenhydramine and amitriptyline (Embryotox Redaktion, f)
Olanzapine	Ask doctor	Readjustment with quetiapine, no change is necessary if drug is indicated (Embryotox Redaktion, h)	Quetiapine, risperidone, haloperidol (Embryotox Redaktion, h)
Opipramol	Ask doctor + stop taking medicine	Readjustment with better-proven antipsychotics, no change is necessary for indications or well-adjusted patients (Embryotox Redaktion, i)	Depending on the indication, e.g. escitalopram for generalised anxiety disorder (Embryotox Redaktion, i)
Quetiapine	Ask doctor + stop taking medicine	Can be prescribed (Embryotox Redaktion, j)	No alternative (Embryotox Redaktion, j)
Risperidone	Ask doctor	Can be prescribed, if necessary, readjustment with quetiapine (Embryotox Redaktion, k)	Quetiapine (Embryotox Redaktion, k)
Moclobemide	Ask doctor	Should not be used in the case of planned pregnancy or new adjustment, in well-adjusted patients, a switch should be avoided if crises are feared (Embryotox Redaktion, g)	Tricyclic antidepressants (amitriptyline, nortriptyline, imipramine) SSRI (citalopram, sertraline) (Embryotox Redaktion, g)
Venlafaxine	Ask doctor + rather stop taking medicine + increased risk of vaginal bleeding	If necessary, readjustment with citalopram or sertraline, no change is necessary for well-adjusted patients (Embryotox Redaktion, m)	Citalopram, sertraline (Embryotox Redaktion, m)
Citalopram	Ask doctor + rather stop taking medicine + increased risk of vaginal bleeding	Drug of choice (Embryotox Redaktion, b)	No alternative (Embryotox Redaktion, b)
Escitalopram	Ask doctor + rather stop taking medicine + increased risk of vaginal bleeding	No change is necessary for well-adjusted patients (Embryotox Redaktion, d)	Citalopram, sertraline (Embryotox Redaktion, d)
Sertraline	Ask doctor + rather stop taking medicine + increased risk of vaginal bleeding	Drug of choice (Embryotox Redaktion, l)	No alternative (Embryotox Redaktion, l)

**Fig. 7:**
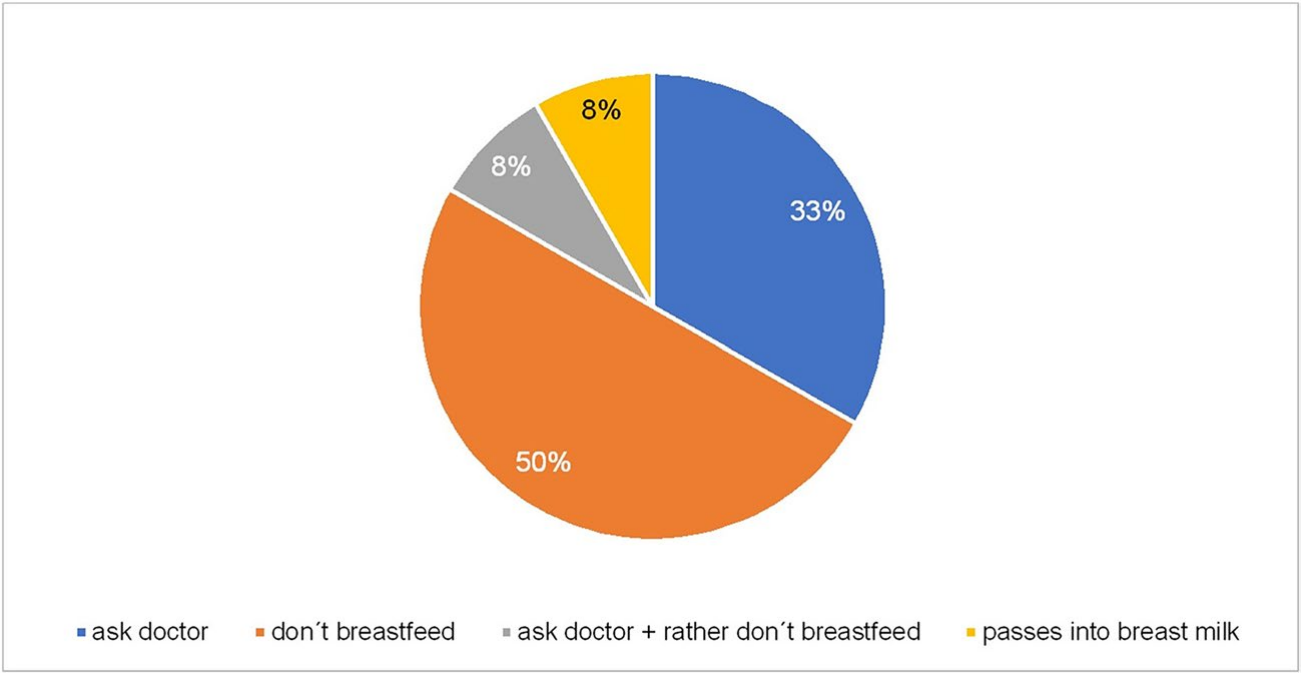
Package insert recommendations during breastfeeding-overall. This diagram shows the distribution of different recommendations given in all 311 PIs for the breastfeeding period

The PIs also provide only vague information for the breastfeeding phase. For the use of haloperidol, risperidone, moclobemide and venlafaxine, the only advice given is to consult a doctor. This recommendation is supplemented in the PI of citalopram by the advice not to breastfeed. A specific ban on breastfeeding is issued for the use of clozapine, melperone, olanzapine, quetiapine and sertraline. The PIs for escitalopram state that the medication can pass into breast milk, but there are no more specific instructions (Fig. 7, Fig. S7). Embryotox gives more differentiated recommendations here. Haloperidol can be taken during breastfeeding in low doses and as monotherapy, but attention should be paid to developmental delays, sedation, drinking difficulties, restlessness, extrapyramidal motor disorders and gastrointestinal symptoms (Embryotox Redaktion, e). The same applies to the use of clozapine, although this is viewed somewhat more critically, as the risk of agranulocytosis in the child cannot be ruled out (Embryotox Redaktion, c). Melperone and opipramol can also be taken during the breastfeeding phase, but here too attention must be paid to possible drinking difficulties, restlessness, extrapyramidal motor disorders and gastrointestinal symptoms (Embryotox Redaktion, f; Embryotox Redaktion, i). Olanzapine, quetiapine, and risperidone are permitted in low doses and in monotherapy, but the newborn should also be closely monitored here (Embryotox Redaktion, h; Embryotox Redaktion, j; Embryotox Redaktion, k). Breastfeeding is also acceptable in the context of antidepressant therapy with moclobemide, venlafaxine, citalopram and escitalopram, provided there are no signs of sedation, drinking difficulties or restlessness (Embryotox Redaktion, b; Embryotox Redaktion, d; Embryotox Redaktion, g; Embryotox Redaktion, m). According to Embryotox, however, the drug of choice during breastfeeding is sertraline (Embryotox Redaktion, l).

**Fig. 8:**
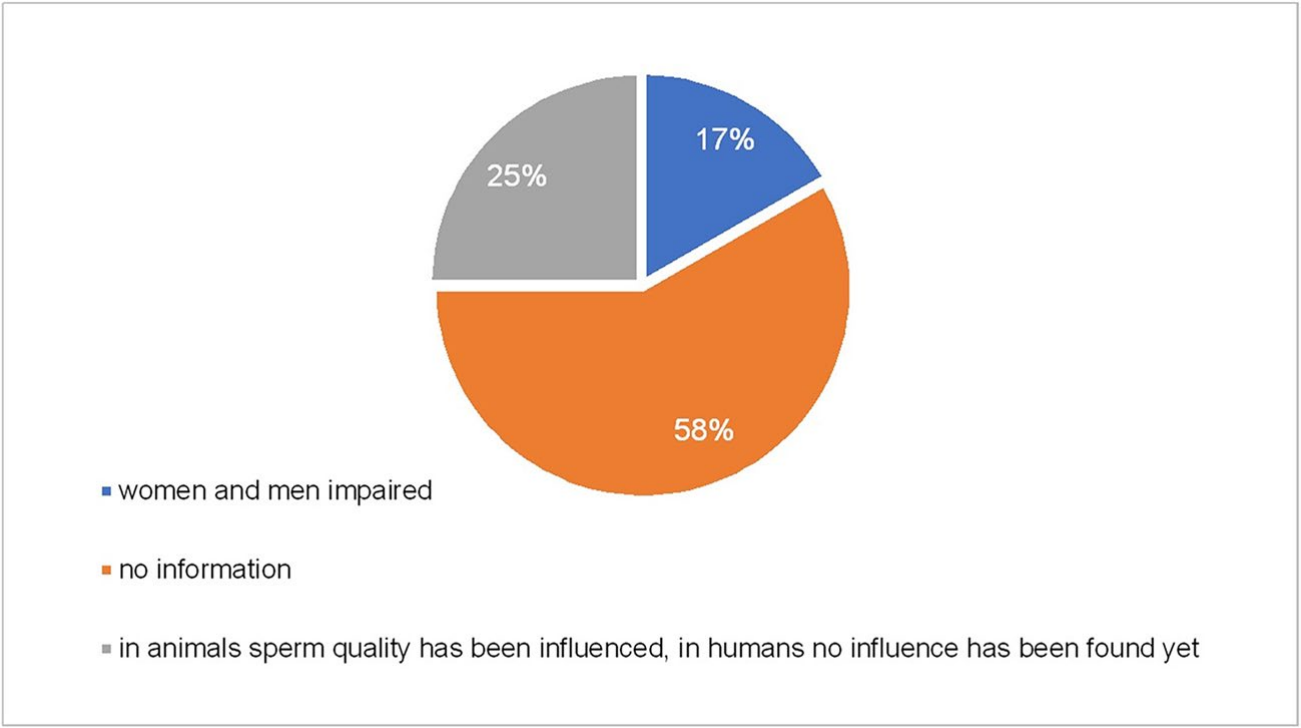
Effects of medication on fertility-overall. This diagram shows the distribution of potential effects of psychotropic drugs on fertility given in all 311 PIs

For a comprehensive overview of the effects of psychotropic drugs on reproduction, the topic of fertility and sexual dysfunction should also be considered (Fig. 8, Fig. S8). However, there is no information on fertility in most PIs. According to the PIs for haloperidol and risperidone, both men and women can be affected by infertility because of taking the drugs. For the drugs citalopram, escitalopram and sertraline, the PIs point out that reduced sperm quality has only been observed in animal studies but has not been proven in humans. Since 2011, various studies have repeatedly suggested that “SSRI treatment can be associated with impaired semen quality” (Elnazer and Baldwin 2014; Relwani et al. 2011). However, a larger study confirmed that “SSRI exposure was not associated with impaired semen parameters” (Pham et al. 2022).

Regarding sexual dysfunction, the PIs provide very different information. Taking moclobemide is not said to increase the risk of sexual dysfunction. Risperidone and venlafaxine can lead to sexual dysfunction, but no further details are given in the package inserts for either medication. Risperidone can lead to reduced sexual satisfaction, ejaculation disorders, and reduced sensitivity of the genitals (van Bruggen et al. 2009). No substantiated data could be found regarding sexual dysfunction after taking venlafaxine. Quetiapine can lead to sexual dysfunction, but to a much lesser extent than when taking other mGPCR antagonists (Park et al. 2012). However, no information is provided in the package inserts in this regard. According to the PI, taking haloperidol can lead to loss of libido, erectile dysfunction, or problems during sexual intercourse. Olanzapine can cause sexual dysfunction in the form of erectile dysfunction and a reduced sex drive. Taking clozapine can lead to erectile and ejaculatory dysfunction (Hummer et al. 1999). According to the PI, melperone may cause problems with sexual arousal, although no substantiated data could be found on this (search keywords on PubMed: melperone AND sexual dysfunction). The PI of opipramol mentions that its use can lead to ejaculation and potency disorders as well as erectile and potency disorders. Potency disorders are associated with the inability to have intercourse with penetration, including erectile dysfunction (Antwerpes 2012). This is therefore an unnecessary, confusing duplication of words. According to the PI, citalopram can possibly lead to changes in libido, ejaculation, and erectile dysfunction, as well as impotence. According to PI, taking escitalopram leads to a reduction in sexual interest, orgasmic disorders, erectile dysfunction, and delayed ejaculation. These symptoms have already been documented in several studies with validated data (Montejo et al. 2015; Perlis et al. 2009). Similar to escitalopram, sertraline can lead to reduced sexual interest and orgasmic disorders, although the latter are not mentioned in the PIs but were documented in one study (Tram et al. 2023).

### Driving ability and the operation of machinery

Taking psychotropic drugs can lead to symptoms such as drowsiness, tiredness, dizziness, vertigo, or visual disturbances. These symptoms occur particularly in the initial phase when the dose is increased or when the preparation is changed and can have a negative effect on attention and reactions. Active participation in road traffic and the operation of machinery may therefore be impaired.

For most substances, the manufacturers agree on which instructions should be followed in this regard. The PIs of haloperidol ask patients to consult their doctor. This is necessary, especially as the effects of haloperidol and risperidone on the ability to drive were already demonstrated in a small study in 2005 (Soyka et al. 2005). If symptoms occur while taking clozapine, participation in road traffic is prohibited. As early as 1999 the influence of clozapine on the ability to drive was documented (Grabe et al. 1999). The PI of olanzapine also warns against driving or advises to consult a doctor beforehand. Patients suffering from schizophrenia exhibit impaired driving ability because of taking olanzapine (Biedermann et al. 2022). According to the package insert, the doctor should decide on the patient’s fitness to drive when taking melperone. The PI of opipramol clearly prohibits participation in road traffic and the operation of machinery. The PI of risperidone also initially prohibits these activities, but this prohibition can be lifted after consultation with the doctor. The driving impairments of risperidone and quetiapine were documented in a controlled study (Brunnauer and Laux 2012). According to PI, the use of quetiapine, venlafaxine, escitalopram, and sertraline should be decided on an individual basis, depending on the effect of the medication. According to a review from 2013, “there is evidence that the SSRIs (citalopram, escitalopram, […] sertraline […]) and the serotonin noradrenaline reuptake inhibitor (SNRI) venlafaxine have no deleterious effects on driving ability” (Brunnauer and Laux 2013). The package inserts for moclobemide, and citalopram contain different information for the same active ingredient from the various manufacturers. Most of the package inserts for citalopram (manufacturer a, c, d, and r) state that an impairment of everyday activities is to be expected and that accordingly no vehicle may be driven without consulting a doctor (identification numbers 251–260 and 262 and 267–270). The other manufacturers b, e, and r state that there should be no impairment, but that attention must still be paid to the individual’s ability to react (identification numbers 261 and 263–266 and 271–274).

In the case of moclobemide, manufacturer a advises against driving and operating machinery, especially in the initial phase of administration (identification numbers 226–227), while according to manufacturer b and c no adverse effects are to be expected, but attention should nevertheless be paid to individual reactions (identification numbers 228–231). According to Ramaekers, “moclobemide [does not] impair driving performance” (Ramaekers et al. 1994).

### Discontinuation symptoms

**Fig. 9:**
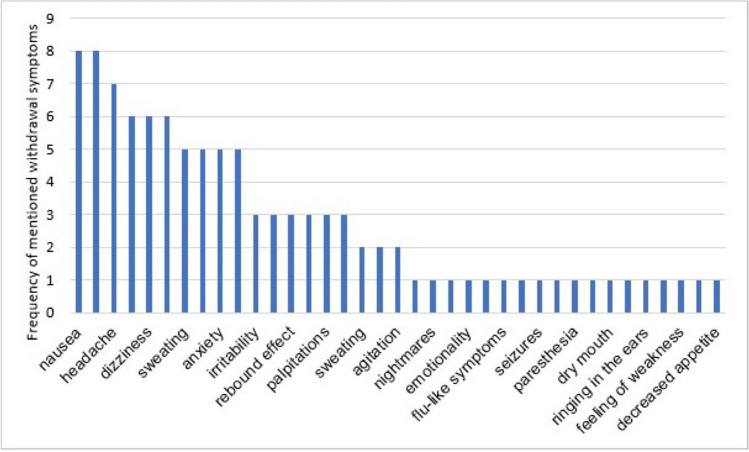
The most frequently mentioned discontinuation symptoms in PIs. This diagram shows a variety of discontinuation symptoms listed according to the number of active substances they are mentioned in

Sudden discontinuation of psychotropic drugs can lead to discontinuation symptoms, according to the PIs. The information in the product information is standardised for most active substances. Figure 9 shows the most frequently mentioned discontinuation symptoms in PIs. For haloperidol, the discontinuation symptoms of nausea, vomiting and sleep disturbances are mentioned in the PI. These could not be substantiated by empirical data. According to the PI, the sudden discontinuation of clozapine can also lead to nausea and vomiting, but also to diarrhoea, sweating, headaches, and the rebound effect. These symptoms have also been described (Blackman and Oloyede 2021). The rebound effect is also mentioned in the PI of risperidone as a discontinuation symptom but has not yet been scientifically proven (search keywords: risperidone AND rebound effect). However, symptoms of tardive dyskinesia have been described, which have not yet been mentioned in the PI (Alhamoud et al. 2022). According to the PI, symptoms such as sweating, tremors, anxiety, nausea, vomiting, and insomnia can occur if olanzapine is suddenly discontinued. This information is also not supported by data (search keywords on PubMed: olanzapine AND discontinuation syndrome). In addition to the symptoms mentioned, hyperthermia can occur when olanzapine is discontinued, which is not mentioned in the package inserts (Zhao et al. 2015).

According to PI, the sudden discontinuation of opipramol can lead to restlessness, sweating, sleep disturbances, nausea, and vomiting, whereby the last two symptoms are only mentioned by manufacturer a (identification numbers 112–113). These symptoms are currently not scientifically proven (search keywords on PubMed: opipramol AND discontinuation syndrome). According to the PI, insomnia, nausea, vomiting, diarrhoea, headache, dizziness and irritability may occur after discontinuation of quetiapine. The discontinuation symptoms “nausea, vomiting, agitation, restlessness, diaphoresis, irritability, anxiety, dysphoria, sleep disturbance, insomnia, tachycardia, hypertension and dizziness” in a case report (Monahan et al. 2021). No information on possible discontinuation symptoms is provided in the PI of melperone (search keywords on PubMed: melperone AND discontinuation syndrome). For the substances moclobemide, venlafaxine, and citalopram, the information provided by the individual manufacturers varies. For moclobemide, manufacturer a states that no discontinuation symptoms occur (identification numbers 226–227), manufacturer b states that a rebound effect may occur (identification numbers 230–231), and manufacturer c mentions the symptoms of sensory disturbances, dizziness, palpitations, visual disturbances, agitation, and confusion (identification numbers 228–229). The latter discontinuation symptoms have also been described in clinical studies (Curtin et al. 2002; Minot et al. 1993). The PIs of venlafaxine all mention the same possible discontinuation symptoms. Only the manufacturer a differs by listing additional symptoms such as hypertension, reduced vision, aggressiveness, and suicidal thoughts (identification numbers 232–234). The symptoms were confirmed (Wang and Cosci 2021). The aspect of suicidality and aggressiveness was first discussed in 2020 as part of a case report, although an actual correlation with discontinuation of treatment has yet to be confirmed (Gahr et al. 2020). Most PIs for citalopram only mention that discontinuation symptoms may occur. Only the manufacturer a explicitly mentions the symptoms of dizziness, sleep disturbances, restlessness, confusion, nausea, vomiting, diarrhoea, headache, and visual disturbances (identification numbers 251–254), which were also mentioned in the literature (Astorne Figari et al. 2014). One discontinuation symptom not mentioned in the PI is sexual dysfunction such as premature ejaculation, described in a case report (Adson and Kotlyar 2003).

The PI of the two NE/5-HT enhancers escitalopram and sertraline show consistent data within the same substance group. Discontinuation of escitalopram can lead to pinprick-like, burning, or electric current-like sensations, but a number of other vegetative and psychological symptoms have also been demonstrated (Yasui-Furukori et al. 2016). The PIs of sertraline cite sensory disturbances, tremors, sleep disturbances, agitation, anxiety, headaches, nausea, vomiting, and dizziness as discontinuation symptoms. These were also mentioned in several publications (Coupland et al. 1996; Zajecka et al. 1997).

### Adverse drug reactions (ADR)

**Fig. 10:**
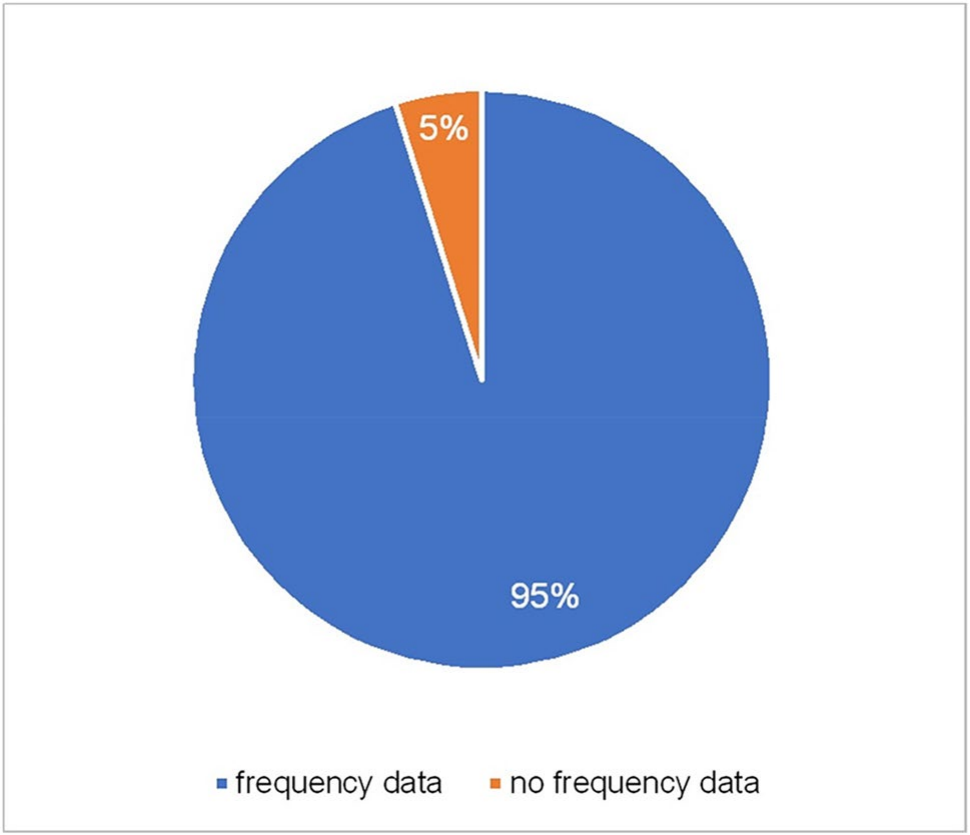
Frequency of ADRs. This diagram shows the distribution between PI with frequency data (blue) and without any frequency data (orange)

Adverse drug reactions are always described in PI as “side effects”, which are listed in a long list. This can lead to a focus on the negative effects, which may be reinforced by the already negative connotation of the term “side effects”. To counteract this, it is necessary to provide frequency classifications. 95% of these are either given directly before the respective list of side effects or in a preceding table. In the remaining 5% of PIs, all for the active substance melperone, no frequency information is provided (Fig. 10). The frequencies are classified into the 5 groups: “very common: may affect more than 1 in 10 people treated”, “common: may affect up to 1 in 10 people treated”, “occasional: may affect up to 1 in 100 people treated”, “rare: may affect up to 1 in 1000 people treated”, “not known: frequency cannot be estimated based on available data”. To highlight the frequency information, most PIs print them in only bold letters or a combination of bold and non-bold letters. The other PIs use underlining, capital letters, or italic as a way to highlight the frequency information; however, it is not as effective and clearly shown.

**Fig. 11:**
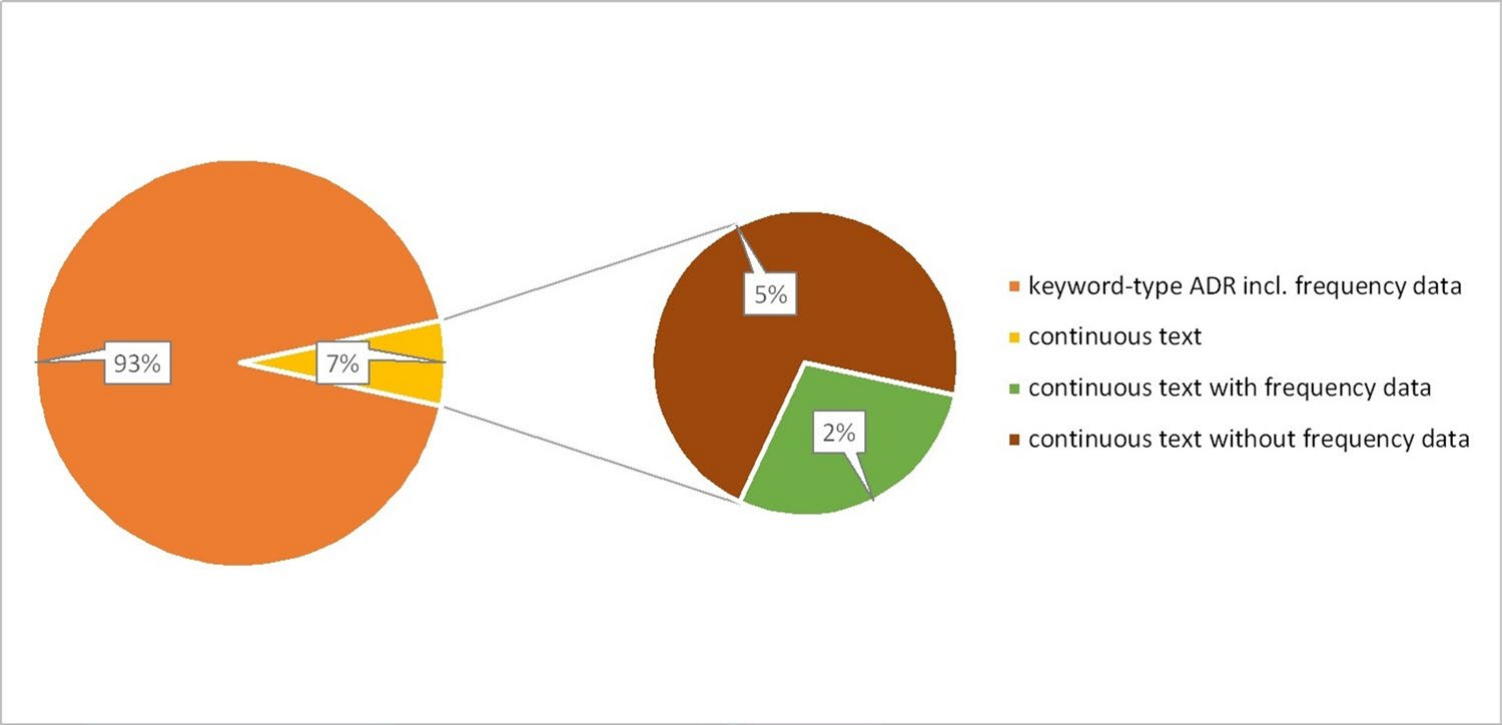
Type of presentation of the ADR and the frequency data. The diagram on the left shows the distribution between PI with keyword-type ADR (orange) and PI with ADR in continuous text. The diagram on the right divides the PI written in continuous text into PI with frequency data (green) and without frequency data (brown)

In addition to the indication of frequencies, a bullet-point list of ADRs is advantageous for a more precise presentation. In 93% of the PIs, these are already written in bullet points; the remaining 7% of the PIs are written in continuous text. It is striking that these PIs also belong exclusively to the active substance melperone. Accordingly, the ADRs for all other active substances analysed are given in bullet points and with frequency information (Fig. 11).

**Fig. 12:**
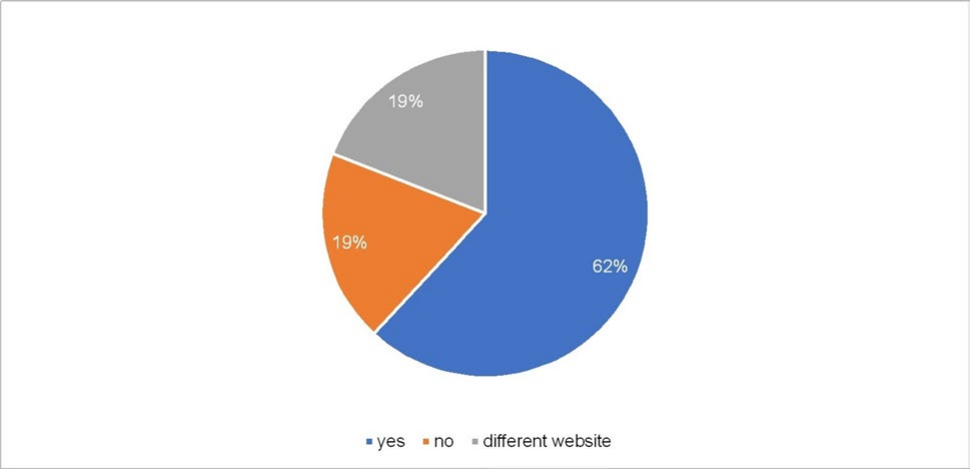
Information on reporting adverse reactions. This diagram shows possible options for reporting adverse reactions. “Yes” (blue) allows the patient to report the adverse reactions on the producer's website. “No” (orange) does not provide the patient with an additional website. “Different website” (grey) means, the PI refers to the website of a different producer

To report further ADRs, this should be reported either to your doctor or pharmacist or on the BfArM website https://www.bfarm.de/DE/Arzneimittel/Pharmakovigilanz/Risiken-melden/_node.html, which is stated on all package inserts. Sixty-two percent of manufacturers also indicate their own website to report further ADRs, 19% of manufacturers indicate the website of another manufacturer, and a further 19% omit this option (Fig. 12).

### Limitations

We are aware that the restriction to certain active ingredients and a maximum of five manufacturers in each case means that only a proportion of all available package inserts was covered. Further drug groups could be added in future analyses. Furthermore, this study was limited to interactions with co-medications that have been known for many years. Interactions with newer active substances that are not yet mentioned in the package insert could not be considered.

## Conclusions

Package inserts serve to provide patients with useful and correct information about the respective medication. Especially in times when many preparations are not available, the importance of this task is growing, as more preparation changes are required. Ideally, the contents of the respective package inserts for an active ingredient should be identical. Despite a standardised legal basis, there are nevertheless numerous variations that make it clear that good and standardised package inserts still have a long way to go. Based on the analysis, the potential for improvement of the PIs of the individual active ingredients was identified and presented in tabular form using the traffic light system (Table 4). Green fields represent a lower need for improvement, a red field represents a high need for improvement. In some cases, a specific improvement aspect is listed in the individual fields.

Concerning the aspects of necessary improvement, standardised information is required in order not to confuse patients unnecessarily. In addition, the information must of course be complete and correct. This applies, for example, to the unproven increased risk of suicide with quetiapine or the withdrawal symptoms not mentioned in the PIs for risperidone, clozapine or olanzapine. Missing information could potentially lead to a careless handling of the medication and could endanger the patient´s life and in the worst case that of other people as well.

In addition to the urgently needed corrective measures mentioned above, there are other ways to optimise the PI. For example, it would make sense to indicate not only the substance group or the mode of action, but both. Particularly in the case of package inserts, which currently only state the group of substances, a supplementary explanation of the mode of action could increase confidence in the medicine. Furthermore, a clearer presentation of interactions with co-medication, withdrawal symptoms and ADRs would be a good idea so that patients can find the necessary information more quickly and reliably.

The particularly important information in the section on pregnancy and breastfeeding has so far been superficial and unfounded. It seems obvious that this information has so far only served to provide legal protection, but not to thoroughly inform the patient. A direct comparison with Embryotox is recommended here, or a reference to the relevant source to provide both the patient and the treating doctor with sufficient information.

Finally, regular updates of the PI are necessary. Interactions with irrelevant drugs must be promptly removed from the package inserts and new interactions with new drugs must be urgently added. Overall, it is necessary for PIs to be updated much more frequently than is currently the case, given the flood of new substances coming onto the market. The legislator is called upon to set intervals at which package inserts must be updated. In future, there should be an up-to-date, complete, correct, clear and comprehensible package insert for every preparation to provide patients with comprehensive information independently of doctors and digital media.

**Table 4:**
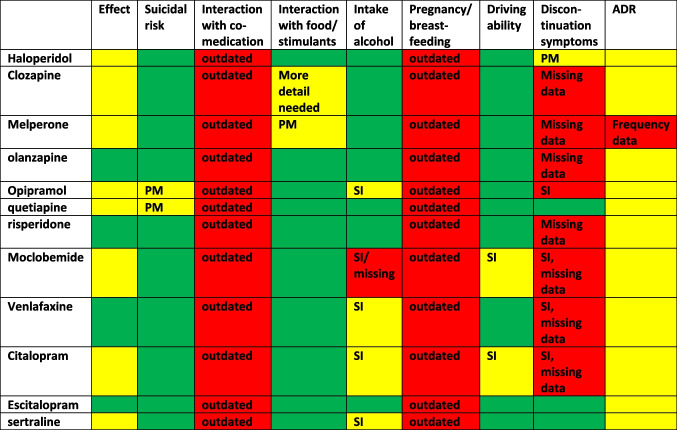
Analysis of the improvement potential of package inserts for individual active ingredients. Red fields indicate a high need for improvement, yellow fields indicate a medium need for improvement, and green fields indicate a lower need for improvement. In some cases, specific aspects for improvement were mentioned (SI = lack of standardised information, PM = does not comply with the information on PubMed)

### Take-home message and future studies

- The package inserts of the individual manufacturers must be revised so that standardised and exclusively correct information is provided. Such measures will reduce confusion among patients and increase their drug adherence.
- The information provided on taking the drug during pregnancy is inadequate, incorrect or out of date. It should be adapted to the current status of the Pharmacovigilance and Advisory Centre for Embryonal Toxicology of the Charité-Universitätsmedizin Berlin (Embryotox).
- The lists of interactions with co-medications are too long and in some cases outdated. They should be shortened and updated.
- In future studies, the PI of all drugs marketed in Germany must be analysed according to this scheme to detect differences in quality.
- In the future, surveys of psychiatric patients on the readability of package inserts should be conducted.

## Supplementary Information

Below is the link to the electronic supplementary material.

Supplementary file1 (DOCX 1656 KB)

## Data Availability

All source data of this study are available from the authors upon reasonable request.

## Abbreviations

PI: Package insert
NE/5-HT-enhancer: Norepinephrine/5-hydroxtryptamine-enhancer
mGPCR antagonist: Antagonists at multiple G-protein-coupled receptors
TCA: Tricyclic antidepressants
SSRI: Selective serotonin reuptake inhibitor
SNRI: Serotonin-noradrenaline reuptake inhibitor
Embryotox: Pharmacovigilance and Advisory Centre for Embryotoxicology
ADR: Adverse drug reactions
BfArM: Bundesinstitut für Arzneimittel und Medizinprodukte
SI: Lack of standardised information
PM: Does not comply with the information on PubMed
