# Supplementary material for: Insufficient correctness of package inserts for psychotropic drugs in Germany
Source: Naunyn Schmiedebergs Arch Pharmacol. 2024 Sep 20;398(3):2879–95. doi: 10.1007/s00210-024-03430-y (PMC11919942; doi:10.1007/s00210-024-03430-y)
Supplement: Supplementary file 1 — Supplementary file1 (DOCX 1656 KB) [file 210_2024_3430_MOESM1_ESM.docx]

**Supplemental Figures S1-S8 and Tables S1 and S2**

**Adina Arning and Roland Seifert**

**Comprehensibility of package inserts for psychotropic drugs**


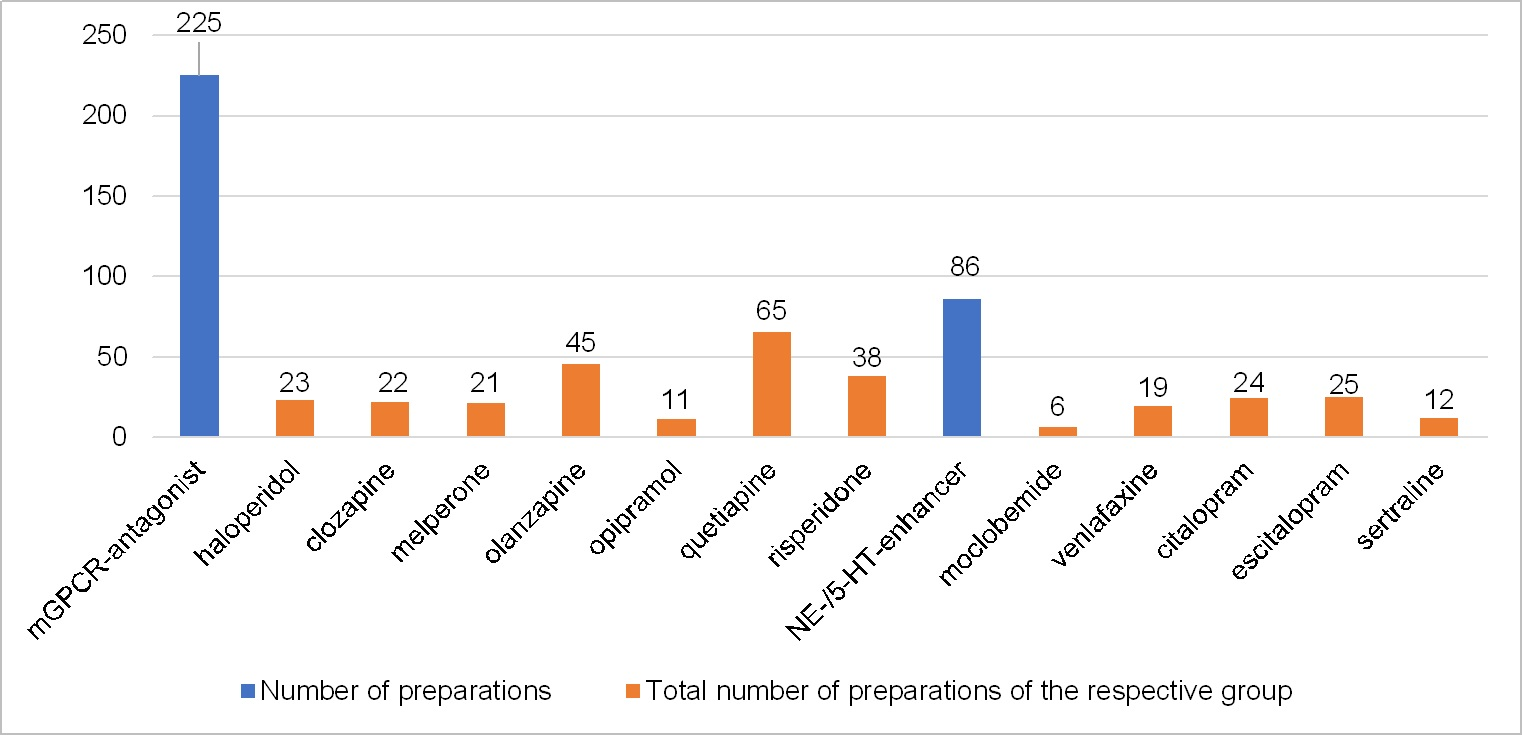


**Fig. S 1.** Distribution of the analysed package inserts between mGPCR-antagonists and NE-/5-HT-enhancer.

**
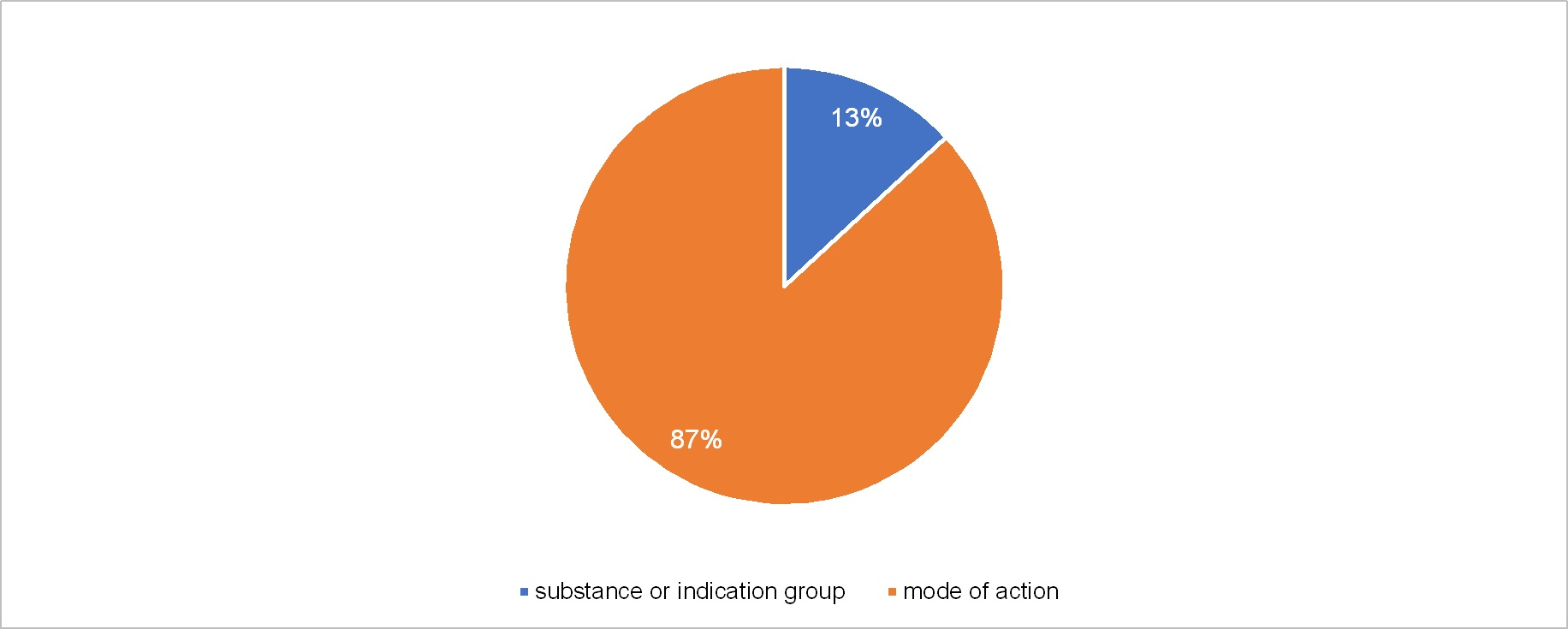
Fig. S 2.** Effect - risperidone. This diagram shows the distribution of all risperidone PI between PI in which the substance or indication group is mentioned (blue) and PI with the mode of action explained (orange).

**
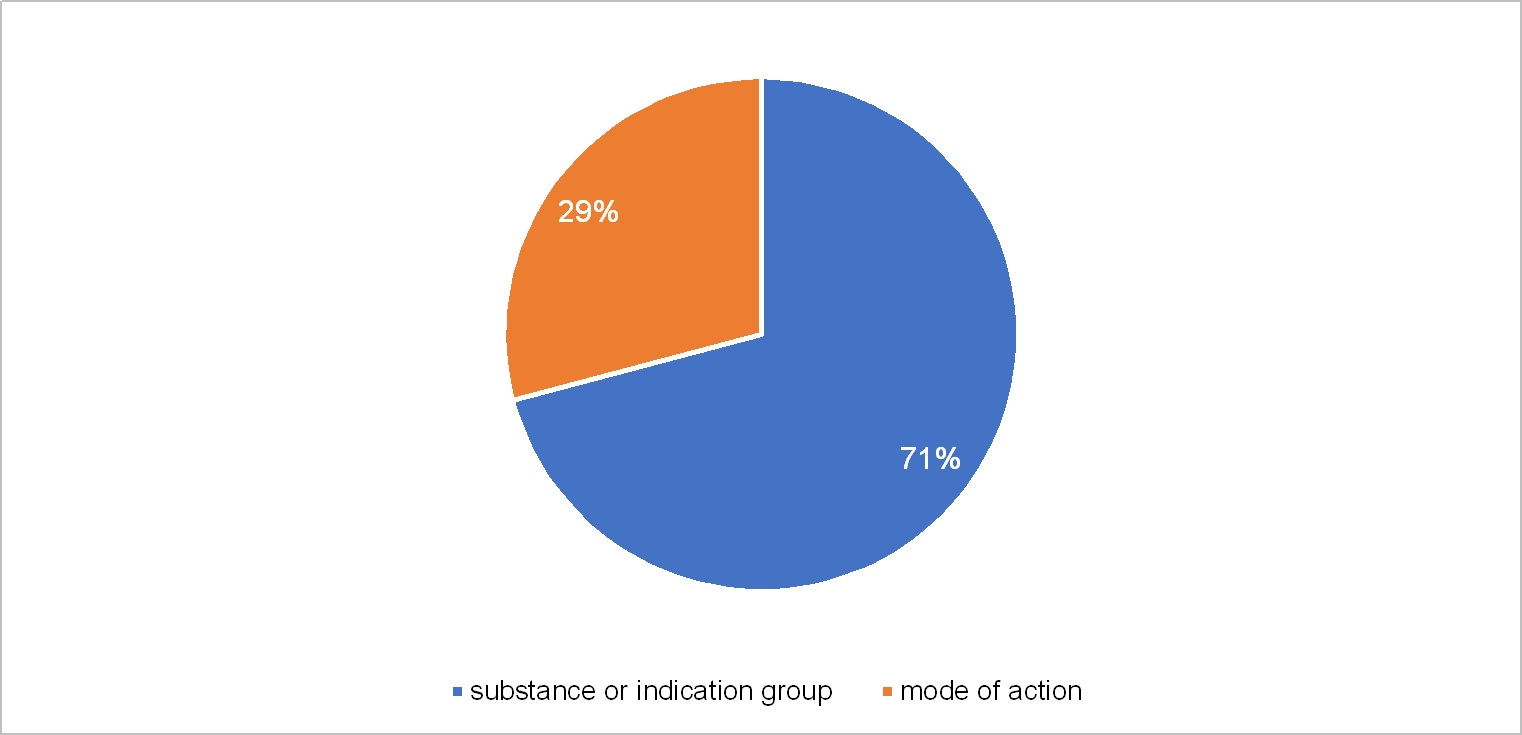
Fig. S 3.** Effect - citalopram. This diagram shows the distribution of all citalopram PI between PI in which the substance or indication group is mentioned (blue) and PI with the mode of action explained (orange).

**
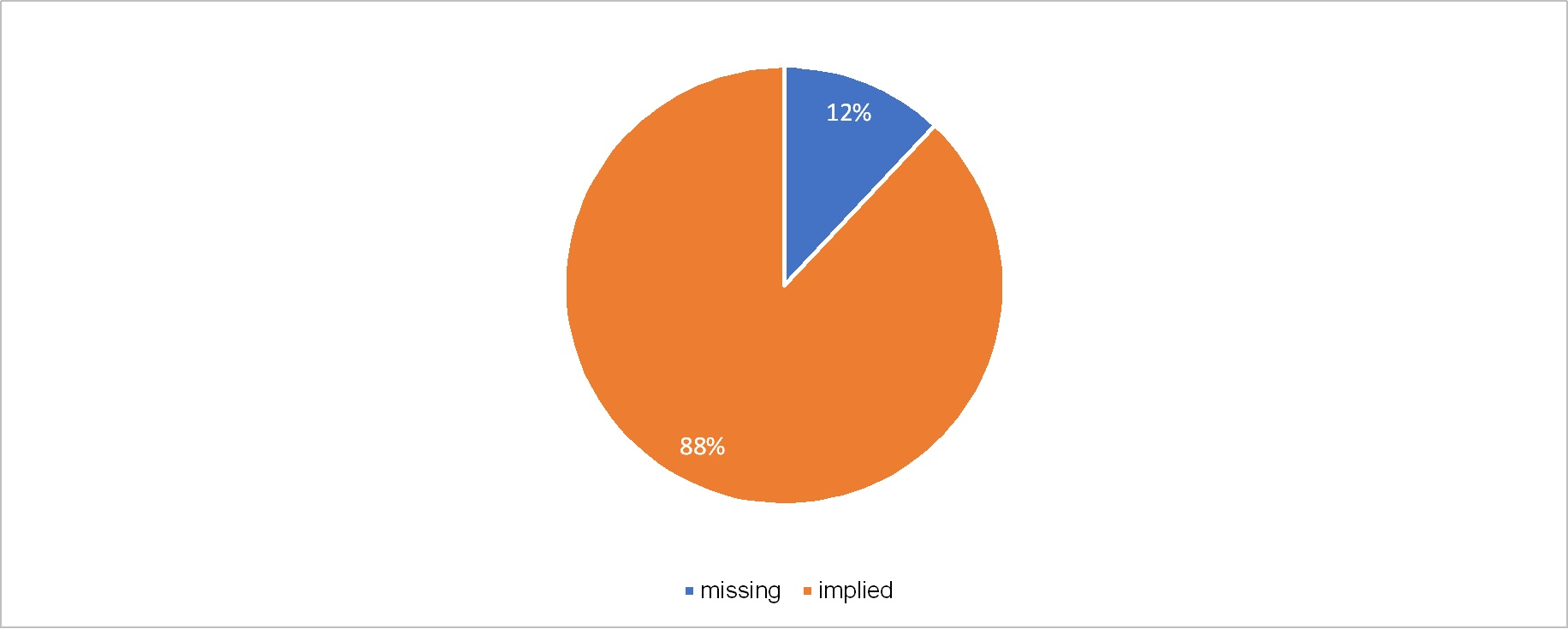
Fig. S 4.** Effect - escitalopram. This diagram shows the distribution of all escitalopram PI between PI in which the substance or indication group is mentioned (blue) and PI with the mode of action explained (orange).


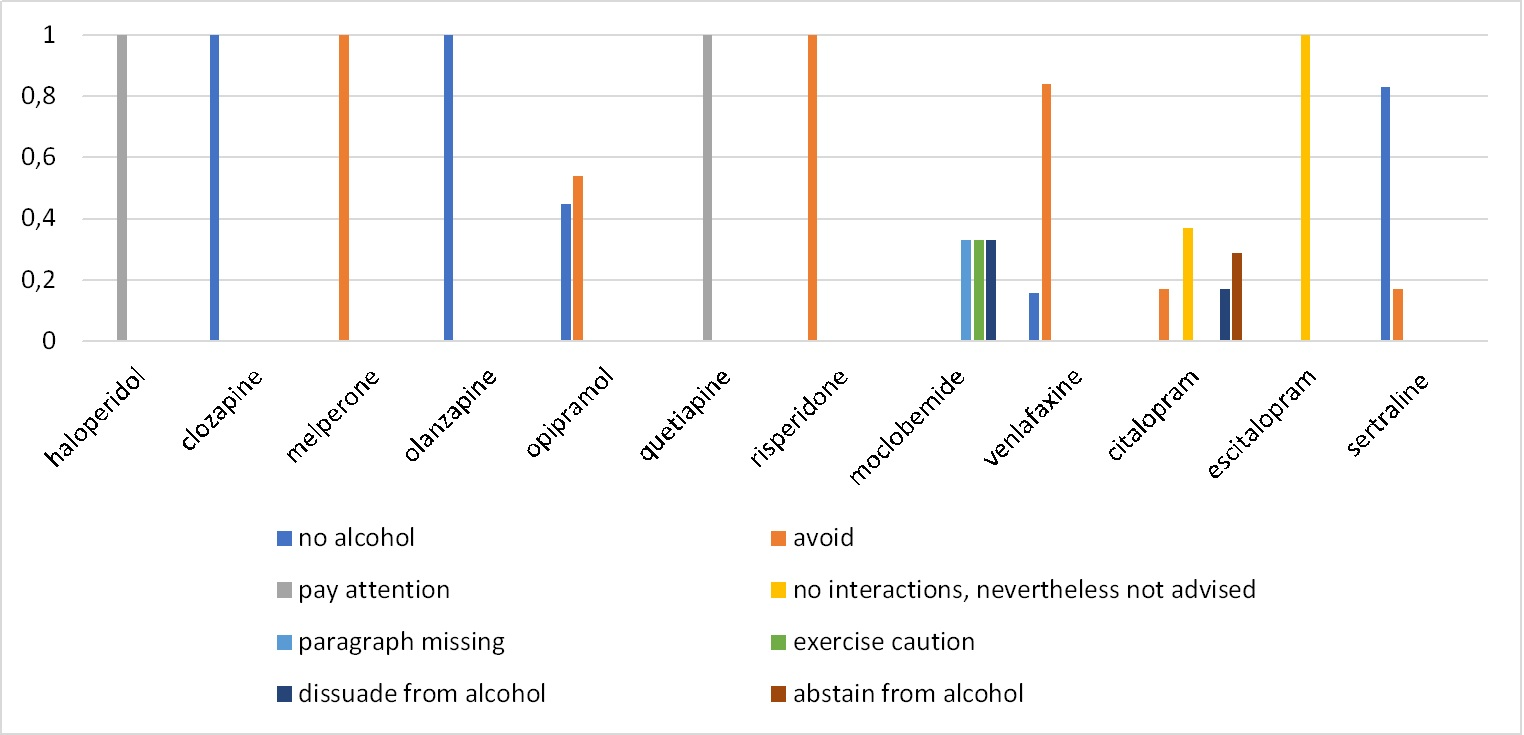


**Fig. S 5. V**arious formulations in the package inserts of the individual drugs that warn about the additional intake of alcohol. This diagram shows the different formulations used to warn about the additional intake of alcohol in the PI of the different substances.


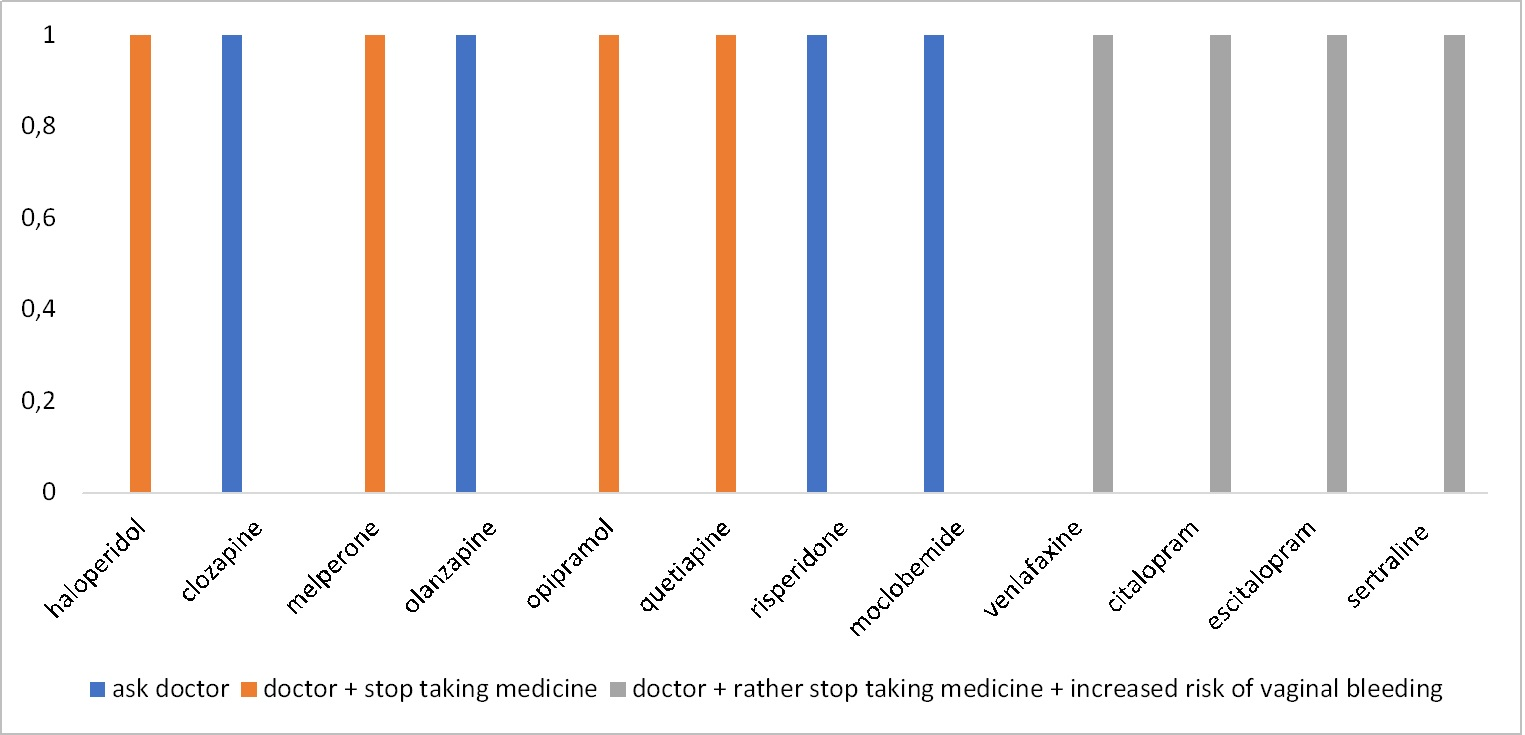


**Fig. S 6.** Package insert recommendations during pregnancy by drug. This diagram shows the different recommendations for the time during the pregnancy given in the PI of the respective drugs.

**
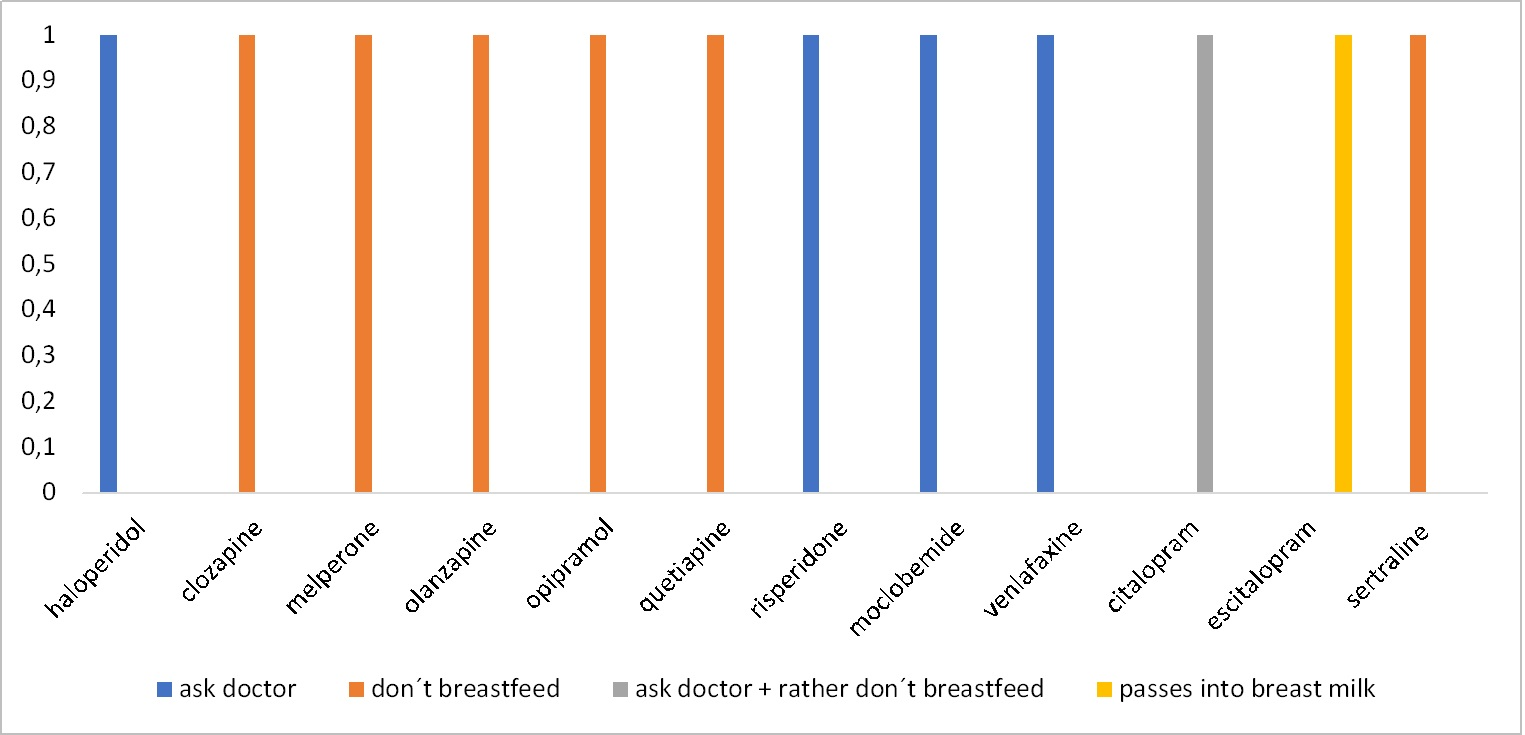
**

**Fig. S 7.** Package insert recommendations during breastfeeding by drug. This diagram shows the different recommendations for the breastfeeding period given in the PI of the respective drugs.

**
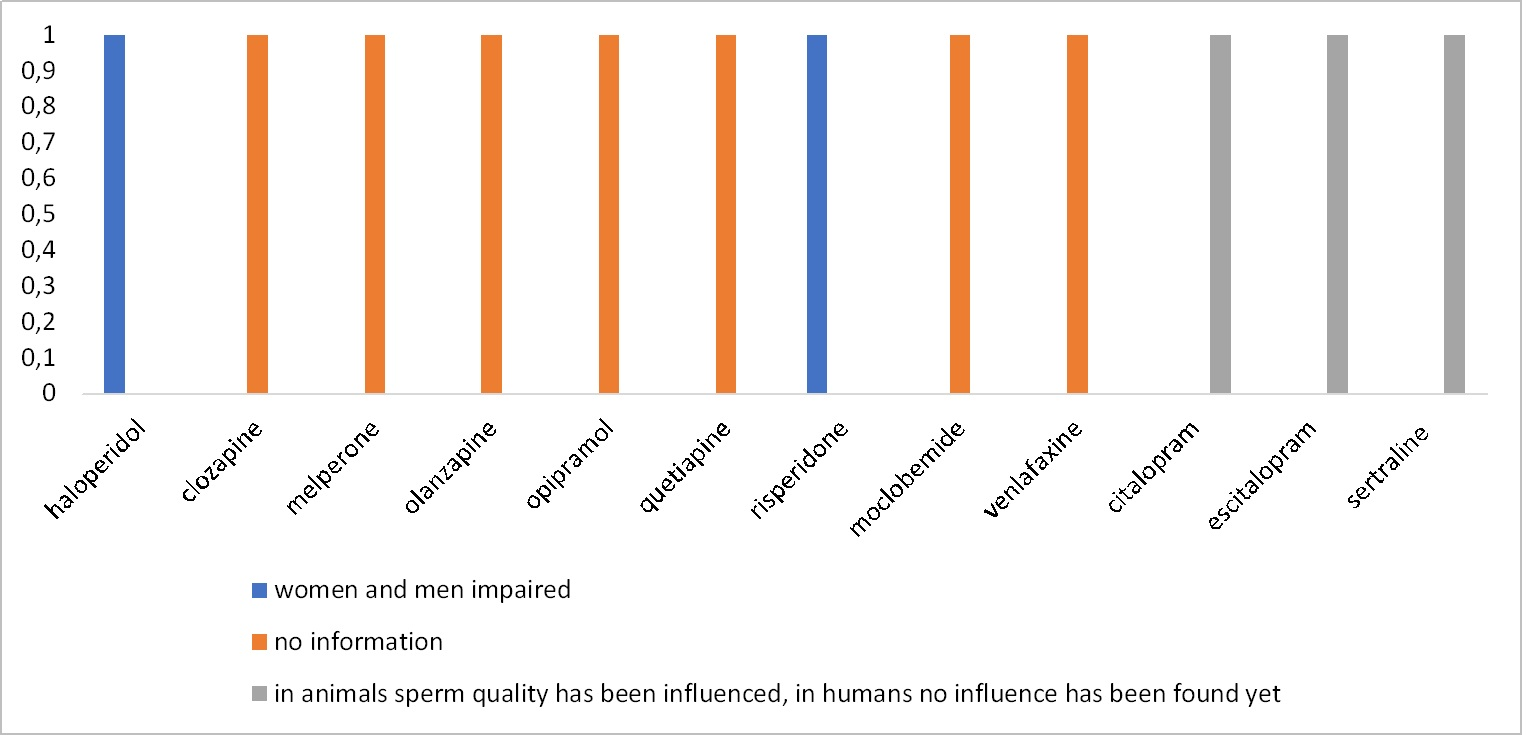
**

**Fig. S 8.** Effects of medication on fertility by drug. This diagram shows the possible effect on fertility of each active substance as mentioned in the PI.

| **Identification number** | **Active substance** | **Manufacturer (coded)** | **Form of application** | **dosage** |
| --- | --- | --- | --- | --- |
| 1 | Haloperidol | a | tablets | 1 mg |
| 2 | Haloperidol | a | tablets | 2 mg |
| 3 | Haloperidol | a | tablets | 5 mg |
| 4 | Haloperidol | a | tablets | 10 mg |
| 5 | Haloperidol | a | injection | 5 mg |
| 6 | Haloperidol | a | drops | 2 mg/ml |
| 7 | Haloperidol | b | tablets | 1 mg |
| 8 | Haloperidol | b | tablets | 4 mg |
| 9 | Haloperidol | b | tablets | 5 mg |
| 10 | Haloperidol | b | tablets | 12 mg |
| 11 | Haloperidol | b | tablets | 20 mg |
| 12 | Haloperidol | b | solution | 2 mg/ml |
| 13 | Haloperidol | b | solution | 10 mg/ml (forte) |
| 14 | Haloperidol | b | injection | 5 mg/ml |
| 15 | Haloperidol | b | injection | 50 mg/ml |
| 16 | Haloperidol | b | injection | 100 mg/ml |
| 17 | Haloperidol | b | injection | 500 mg/10 ml |
| 18 | Haloperidol | k | tablets | 1 mg |
| 19 | Haloperidol | k | tablets | 5 mg |
| 20 | Haloperidol | k | tablets | 10 mg |
| 21 | Haloperidol | k | solution | 2 mg/ml |
| 22 | Haloperidol | k | injection | 5 mg/ml |
| 23 | Haloperidol | k | injection | 50 mg/ml |
| 24 | Clozapine | a | tablets | 100 mg |
| 25 | Clozapine | a | tablets | 200 mg |
| 26 | Clozapine | c | tablets | 25 mg |
| 27 | Clozapine | c | tablets | 50 mg |
| 28 | Clozapine | c | tablets | 100 mg |
| 29 | Clozapine | c | tablets | 200 mg |
| 30 | Clozapine | f | tablets | 25 mg |
| 31 | Clozapine | f | tablets | 50 mg |
| 32 | Clozapine | f | tablets | 100 mg |
| 33 | Clozapine | f | tablets | 200 mg |
| 34 | Clozapine | j | tablets | 25 mg |
| 35 | Clozapine | j | tablets | 50 mg |
| 36 | Clozapine | j | tablets | 100 mg |
| 37 | Clozapine | j | tablets | 200 mg |
| 38 | Clozapine | s | tablets | 25 mg |
| 39 | Clozapine | s | tablets | 50 mg |
| 40 | Clozapine | s | tablets | 100 mg |
| 41 | Clozapine | s | tablets | 200 mg |
| 42 | Clozapine | b | tablets | 25 mg |
| 43 | Clozapine | b | tablets | 50 mg |
| 44 | Clozapine | b | tablets | 100mg |
| 45 | Clozapine | b | tablets | 200 mg |
| 46 | Melperone | a | tablets | 25 mg |
| 47 | Melperone | a | tablets | 50 mg |
| 48 | Melperone | a | tablets | 100 mg |
| 49 | Melperone | a | tablets | 25 mg/5 ml |
| 50 | Melperone | c | tablets | 25 mg |
| 51 | Melperone | c | tablets | 50 mg |
| 52 | Melperone | c | solution | 25 mg/5 ml |
| 53 | Melperone | d | tablets | 25 mg |
| 54 | Melperone | d | tablets | 100 mg |
| 55 | Melperone | d | solution | 25 mg/5 ml |
| 56 | Melperone | e | tablets | 10 mg |
| 57 | Melperone | e | tablets | 25 mg |
| 58 | Melperone | e | tablets | 50 mg |
| 59 | Melperone | e | tablets | 100 mg |
| 60 | Melperone | e | solution | 25 mg/5 ml |
| 61 | Melperone | b | tablets | 10 mg |
| 62 | Melperone | b | tablets | 25 mg |
| 63 | Melperone | b | tablets | 50 mg |
| 64 | Melperone | b | tablets | 100 mg |
| 65 | Melperone | b | solution | 25 mg/5 ml |
| 66 | Melperone | b | solution liquidum | 25 mg/5 ml |
| 67 | Olanzapine | a | tablets | 2.5 mg |
| 68 | Olanzapine | a | tablets | 5 mg |
| 69 | Olanzapine | a | tablets | 7.5 mg |
| 70 | Olanzapine | a | tablets | 10 mg |
| 71 | Olanzapine | a | tablets | 15 mg |
| 72 | Olanzapine | a | tablets | 20 mg |
| 73 | Olanzapine | a | melting tablets | 5 mg |
| 74 | Olanzapine | a | melting tablets | 10 mg |
| 75 | Olanzapine | a | melting tablets | 15 mg |
| 76 | Olanzapine | a | melting tablets | 20 mg |
| 77 | Olanzapine | c | tablets | 2.5 mg |
| 78 | Olanzapine | c | tablets | 7.5 mg |
| 79 | Olanzapine | c | tablets | 10 mg |
| 80 | Olanzapine | c | tablets | 15 mg |
| 81 | Olanzapine | c | tablets | 20 mg |
| 82 | Olanzapine | c | melting tablets | 5 mg |
| 83 | Olanzapine | c | melting tablets | 15 mg |
| 84 | Olanzapine | h | tablets | 2.5 mg |
| 85 | Olanzapine | h | tablets | 5 mg |
| 86 | Olanzapine | h | tablets | 7.5 mg |
| 87 | Olanzapine | h | tablets | 10 mg |
| 88 | Olanzapine | h | melting tablets | 15 mg |
| 89 | Olanzapine | h | melting tablets | 20 mg |
| 90 | Olanzapine | j | tablets | 2,5 mg |
| 91 | Olanzapine | j | tablets | 5 mg |
| 92 | Olanzapine | j | tablets | 7.5 mg |
| 93 | Olanzapine | j | tablets | 10 mg |
| 94 | Olanzapine | j | tablets | 15 mg |
| 95 | Olanzapine | j | tablets | 20 mg |
| 96 | Olanzapine | b | tablets | 2,5 mg |
| 97 | Olanzapine | b | tablets | 5 mg |
| 98 | Olanzapine | b | tablets | 7.5 mg |
| 99 | Olanzapine | b | tablets | 10 mg |
| 100 | Olanzapine | b | tablets | 15 mg |
| 101 | Olanzapine | b | tablets | 20 mg |
| 102 | Olanzapine | i | tablets | 2.5 mg |
| 103 | Olanzapine | i | tablets | 5 mg |
| 104 | Olanzapine | i | tablets | 7.5 mg |
| 105 | Olanzapine | i | tablets | 10 mg |
| 106 | Olanzapine | i | tablets | 15 mg |
| 107 | Olanzapine | i | tablets | 20 mg |
| 108 | Olanzapine | i | melting tablets | 5 mg |
| 109 | Olanzapine | i | melting tablets | 10 mg |
| 110 | Olanzapine | i | melting tablets | 15 mg |
| 111 | Olanzapine | i | melting tablets | 20 mg |
| 112 | Opipramol | a | tablets | 50 mg |
| 113 | Opipramol | a | tablets | 100 mg |
| 114 | Opipramol | c | tablets | 50 mg |
| 115 | Opipramol | c | tablets | 100 mg |
| 116 | Opipramol | d | tablets | 50 mg |
| 117 | Opipramol | d | tablets | 100 mg |
| 118 | Opipramol | b | tablets | 50 mg |
| 119 | Opipramol | b | tablets | 100 mg |
| 120 | Opipramol | b | tablets | 150 mg |
| 121 | Opipramol | m | tablets | 50 mg |
| 122 | Opipramol | m | tablets | 100 mg |
| 123 | Quetiapine | a | tablets | 25 mg |
| 124 | Quetiapine | a | tablets | 100 mg |
| 125 | Quetiapine | a | tablets | 150 mg |
| 126 | Quetiapine | a | tablets | 200 mg |
| 127 | Quetiapine | a | tablets | 300 mg |
| 128 | Quetiapine | a | retard tablets | 50 mg |
| 129 | Quetiapine | a | retard tablets | 150 mg |
| 130 | Quetiapine | a | retard tablets | 200 mg |
| 131 | Quetiapine | a | retard tablets | 300 mg |
| 132 | Quetiapine | a | retard tablets | 400 mg |
| 133 | Quetiapine | c | tablets | 25 mg |
| 134 | Quetiapine | c | tablets | 50 mg |
| 135 | Quetiapine | c | tablets | 100 mg |
| 136 | Quetiapine | c | tablets | 150 mg |
| 137 | Quetiapine | c | tablets | 200 mg |
| 138 | Quetiapine | c | tablets | 300 mg |
| 139 | Quetiapine | c | tablets | 400 mg |
| 140 | Quetiapine | c | retard tablets | 50 mg |
| 141 | Quetiapine | c | retard tablets | 150 mg |
| 142 | Quetiapine | c | retard tablets | 200 mg |
| 143 | Quetiapine | c | retard tablets | 300 mg |
| 144 | Quetiapine | c | retard tablets | 400 mg |
| 145 | Quetiapine | c | retard tablets | 600 mg |
| 146 | Quetiapine | f | tablets | 25 mg |
| 147 | Quetiapine | f | tablets | 100 mg |
| 148 | Quetiapine | f | tablets | 200 mg |
| 149 | Quetiapine | f | tablets | 300 mg |
| 150 | Quetiapine | f | retard tablets | 50 mg |
| 151 | Quetiapine | f | retard tablets | 100 mg |
| 152 | Quetiapine | f | retard tablets | 150 mg |
| 153 | Quetiapine | f | retard tablets | 200 mg |
| 154 | Quetiapine | f | retard tablets | 300 mg |
| 155 | Quetiapine | f | retard tablets | 400 mg |
| 156 | Quetiapine | g | tablets | 25 mg |
| 157 | Quetiapine | g | tablets | 100 mg |
| 158 | Quetiapine | g | tablets | 150 mg |
| 159 | Quetiapine | g | tablets | 200 mg |
| 160 | Quetiapine | g | tablets | 300 mg |
| 161 | Quetiapine | g | retard tablets | 50 mg |
| 162 | Quetiapine | g | retard tablets | 150 mg |
| 163 | Quetiapine | g | retard tablets | 200 mg |
| 164 | Quetiapine | g | retard tablets | 300 mg |
| 165 | Quetiapine | g | retard tablets | 400 mg |
| 166 | Quetiapine | d | tablets | 25 mg |
| 167 | Quetiapine | d | tablets | 100 mg |
| 168 | Quetiapine | d | tablets | 200 mg |
| 169 | Quetiapine | d | tablets | 300 mg |
| 170 | Quetiapine | d | retard tablets | 50 mg |
| 171 | Quetiapine | d | retard tablets | 150 mg |
| 172 | Quetiapine | d | retard tablets | 200 mg |
| 173 | Quetiapine | d | retard tablets | 300 mg |
| 174 | Quetiapine | d | retard tablets | 400 mg |
| 175 | Quetiapine | b | tablets | 25 mg |
| 176 | Quetiapine | b | tablets | 50 mg |
| 177 | Quetiapine | b | tablets | 100 mg |
| 178 | Quetiapine | b | tablets | 150 mg |
| 179 | Quetiapine | b | tablets | 200 mg |
| 180 | Quetiapine | b | tablets | 300 mg |
| 181 | Quetiapine | b | tablets | 400 mg |
| 182 | Quetiapine | b | retard tablets | 50 mg |
| 183 | Quetiapine | b | retard tablets | 150 mg |
| 184 | Quetiapine | b | retard tablets | 200 mg |
| 185 | Quetiapine | b | retard tablets | 300 mg |
| 186 | Quetiapine | b | retard tablets | 400 mg |
| 187 | Quetiapine | b | retard tablets | 600 mg |
| 188 | Risperidone | a | injection | 25 mg |
| 189 | Risperidone | a | injection | 37.5 mg |
| 190 | Risperidone | a | injection | 50 mg |
| 191 | Risperidone | c | tablets | 0,25 mg |
| 192 | Risperidone | c | tablets | 0.5 mg |
| 193 | Risperidone | c | tablets | 1 mg |
| 194 | Risperidone | c | tablets | 2 mg |
| 195 | Risperidone | c | tablets | 3 mg |
| 196 | Risperidone | c | tablets | 4 mg |
| 197 | Risperidone | c | tablets | 6 mg |
| 198 | Risperidone | c | solution | 1 mg/ml |
| 199 | Risperidone | d | tablets | 0.5 mg |
| 200 | Risperidone | d | tablets | 1 mg |
| 201 | Risperidone | d | tablets | 2 mg |
| 202 | Risperidone | d | tablets | 3 mg |
| 203 | Risperidone | d | tablets | 4 mg |
| 204 | Risperidone | d | tablets | 6 mg |
| 205 | Risperidone | d | solution | 1 mg/ml |
| 206 | Risperidone | e | tablets | 0.25 mg |
| 207 | Risperidone | e | tablets | 0.5 mg |
| 208 | Risperidone | e | tablets | 1 mg |
| 209 | Risperidone | e | tablets | 2 mg |
| 210 | Risperidone | e | tablets | 3 mg |
| 211 | Risperidone | e | tablets | 4 mg |
| 212 | Risperidone | e | tablets | 6 mg |
| 213 | Risperidone | e | tablets | 8 mg |
| 214 | Risperidone | e | solution | 1 mg/ml |
| 215 | Risperidone | o | tablets | 0.5 mg |
| 216 | Risperidone | o | tablets | 1 mg |
| 217 | Risperidone | o | tablets | 2 mg |
| 218 | Risperidone | o | tablets | 3 mg |
| 219 | Risperidone | o | tablets | 4 mg |
| 220 | Risperidone | o | tablets | 6 mg |
| 221 | Risperidone | u | tablets | 0,5 mg |
| 222 | Risperidone | u | tablets | 1 mg |
| 223 | Risperidone | u | tablets | 2 mg |
| 224 | Risperidone | u | tablets | 3 mg |
| 225 | Risperidone | u | tablets | 4 mg |
| 226 | Moclobemide | a | tablets | 150 mg |
| 227 | Moclobemide | a | tablets | 300 mg |
| 228 | Moclobemide | c | tablets | 150 mg |
| 229 | Moclobemide | c | tablets | 300 mg |
| 230 | Moclobemide | b | tablets | 150 mg |
| 231 | Moclobemide | b | tablets | 300 mg |
| 232 | Venlafaxine | a | hard capsules | 37.5 mg |
| 233 | Venlafaxine | a | hard capsules | 75 mg |
| 234 | Venlafaxine | a | hard capsules | 150 mg |
| 235 | Venlafaxine | n | hard capsules | 37.5 mg |
| 236 | Venlafaxine | n | hard capsules | 75 mg |
| 237 | Venlafaxine | n | hard capsules | 150 mg |
| 238 | Venlafaxine | d | hard capsules | 37.5 mg |
| 239 | Venlafaxine | d | hard capsules | 75 mg |
| 240 | Venlafaxine | d | hard capsules | 225 mg |
| 241 | Venlafaxine | e | hard capsules | 37.5 mg |
| 242 | Venlafaxine | e | hard capsules | 75 mg |
| 243 | Venlafaxine | e | hard capsules | 150 mg |
| 244 | Venlafaxine | e | hard capsules | 225 mg |
| 245 | Venlafaxine | b | tablets | 75 mg |
| 246 | Venlafaxine | b | retard tablets | 37.5 mg |
| 247 | Venlafaxine | b | retard tablets | 75 mg |
| 248 | Venlafaxine | b | retard tablets | 150 mg |
| 249 | Venlafaxine | b | retard tablets | 225 mg |
| 250 | Venlafaxine | b | retard tablets | 300 mg |
| 251 | Citalopram | a | tablets | 10 mg |
| 252 | Citalopram | a | tablets | 20 mg |
| 253 | Citalopram | a | tablets | 30 mg |
| 254 | Citalopram | a | tablets | 40 mg |
| 255 | Citalopram | c | tablets | 10 mg |
| 256 | Citalopram | c | tablets | 20 mg |
| 257 | Citalopram | c | tablets | 30 mg |
| 258 | Citalopram | c | tablets | 40 mg |
| 259 | Citalopram | r | tablets | 10 mg |
| 260 | Citalopram | r | tablets | 20 mg |
| 261 | Citalopram | r | tablets | 30 mg |
| 262 | Citalopram | r | tablets | 40 mg |
| 263 | Citalopram | b | tablets | 10 mg |
| 264 | Citalopram | b | tablets | 20 mg |
| 265 | Citalopram | b | tablets | 30 mg |
| 266 | Citalopram | b | tablets | 40 mg |
| 267 | Citalopram | d | tablets | 10 mg |
| 268 | Citalopram | d | tablets | 20 mg |
| 269 | Citalopram | d | tablets | 30 mg |
| 270 | Citalopram | d | tablets | 40 mg |
| 271 | Citalopram | e | tablets | 10 mg |
| 272 | Citalopram | e | tablets | 20 mg |
| 273 | Citalopram | e | tablets | 30 mg |
| 274 | Citalopram | e | tablets | 40 mg |
| 275 | Escitalopram | a | tablets | 5 mg |
| 276 | Escitalopram | a | tablets | 10 mg |
| 277 | Escitalopram | a | tablets | 15 mg |
| 278 | Escitalopram | a | tablets | 20 mg |
| 279 | Escitalopram | a | drops | 20 mg/ml |
| 280 | Escitalopram | f | tablets | 5 mg |
| 281 | Escitalopram | f | tablets | 10 mg |
| 282 | Escitalopram | f | tablets | 15 mg |
| 283 | Escitalopram | f | tablets | 20 mg |
| 284 | Escitalopram | f | drops | 20 mg/ml |
| 285 | Escitalopram | i | tablets | 5 mg |
| 286 | Escitalopram | i | tablets | 10 mg |
| 287 | Escitalopram | i | tablets | 15 mg |
| 288 | Escitalopram | i | tablets | 20 mg |
| 289 | Escitalopram | j | tablets | 5 mg |
| 290 | Escitalopram | j | tablets | 10 mg |
| 291 | Escitalopram | j | tablets | 15 mg |
| 292 | Escitalopram | j | tablets | 20 mg |
| 293 | Escitalopram | l | tablets | 10 mg |
| 294 | Escitalopram | l | tablets | 20 mg |
| 295 | Escitalopram | l | drops | 20 mg/ml |
| 296 | Escitalopram | t | tablets | 5 mg |
| 297 | Escitalopram | t | tablets | 10 mg |
| 298 | Escitalopram | t | tablets | 15 mg |
| 299 | Escitalopram | t | tablets | 20 mg |
| 300 | Sertraline | a | tablets | 50 mg |
| 301 | Sertraline | a | tablets | 100 mg |
| 302 | Sertraline | c | tablets | 50 mg |
| 303 | Sertraline | c | tablets | 100 mg |
| 304 | Sertraline | g | tablets | 50 mg |
| 305 | Sertraline | g | tablets | 100 mg |
| 306 | Sertraline | p | tablets | 50 mg |
| 307 | Sertraline | p | tablets | 100 mg |
| 308 | Sertraline | h | tablets | 50 mg |
| 309 | Sertraline | h | tablets | 100 mg |
| 310 | Sertraline | q | tablets | 50 mg |
| 311 | Sertraline | q | tablets | 100 mg |

**Table S 1.** Assignment of identification numbers to the respective preparation with coded manufacturer, application form and dosage

| **Drug** | **DDD in thousand 2021** | **DDD in thousand 2022** |
| --- | --- | --- |
| Astemizole | -- | -- |
| Benzatropine | -- | -- |
| Bepridil | -- | -- |
| Quinidine | -- | -- |
| Quinine | -- | -- |
| Chloramphenicol | 0.0 | 0.0 |
| Chlorpromazine | -- | -- |
| Desipramine | -- | -- |
| Dextromethorphan | -- | -- |
| Dextropropoxyphene | -- | -- |
| Dipyridamole | -- | -- |
| Disopyramide | -- | -- |
| Dofetilide | -- | -- |
| Dolasetron | -- | -- |
| Ephedrine | -- | -- |
| Guanethidine | -- | -- |
| Halofantrine | -- | -- |
| Ibutilide | -- | -- |
| Indinavir | -- | -- |
| Iproniazid | -- | -- |
| Isocarboxazid | -- | -- |
| Ketoconazole | 0.0 | 0.0 |
| Lisuride | -- | -- |
| Mefloquine | -- | -- |
| Mivacurium | -- | -- |
| Nefazodon | -- | -- |
| Nialamide | -- | -- |
| Pentamidine | 0.0 | 0.0 |
| Pentazocine | -- | -- |
| Pethidine | 0.0 | 0.0 |
| Phenelicin | - | -- |
| Phenindione | -- | -- |
| Phenylbutazone | -- | -- |
| Phenylephrine | 0.0 | 0.0 |
| Phenylpropanolamine | -- | -- |
| Pimozide | 0.0 | 0.0 |
| Procainamide | 0.0 | 0.0 |
| Prochlorperazine | -- | -- |
| Promazine | -- | -- |
| Pseudoephedrine | -- | -- |
| Ranitidine | no longer authorised | no longer authorised |
| Saquinavir | -- | -- |
| Sertindol | 0.0 | 0.0 |
| Sibutramine | -- | -- |
| Sparfloxacin | -- | -- |
| Suxamethonium | -- | -- |
| Telaprevir | -- | -- |
| Telithromycin | -- | -- |
| Thioridazine | 0.0 | 0.0 |
| Ticlopidine | 0.0 | 0.0 |
| Tolbutamide | -- | -- |
| Toremifene | -- | -- |
| Trifluoperazine | -- | -- |
| Triflupromazine | -- | -- |
| Trypotphan | -- | -- |

**Table S 2.** in PIs mentioned, but largely irrelevant drugs. Concrete prescription numbers (DDD) according to the PharMaAnalyst-Wido are listed (Wissenschaftliches Institut der AOK).
